# Recent Advances in the Development of Semisynthetic Glycopeptide Antibiotics: 2014–2022

**DOI:** 10.1021/acsinfecdis.2c00253

**Published:** 2022-07-27

**Authors:** Emma van Groesen, Paolo Innocenti, Nathaniel I. Martin

**Affiliations:** Biological Chemistry Group, Institute of Biology Leiden, Leiden University 2333 BE Leiden, The Netherlands

**Keywords:** vancomycin, glycopeptides, antibiotic resistance, semisynthesis, mechanism of action

## Abstract

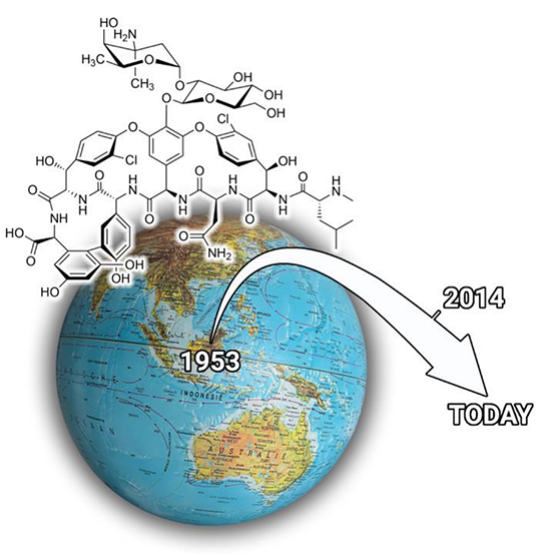

The accelerated appearance of drug-resistant bacteria poses an ever-growing threat to modern medicine’s capacity to fight infectious diseases. Gram-positive species such as methicillin-resistant *Staphylococcus aureus* (MRSA) and *Streptococcus pneumoniae* continue to contribute significantly to the global burden of antimicrobial resistance. For decades, the treatment of serious Gram-positive infections relied upon the glycopeptide family of antibiotics, typified by vancomycin, as a last line of defense. With the emergence of vancomycin resistance, the semisynthetic glycopeptides telavancin, dalbavancin, and oritavancin were developed. The clinical use of these compounds is somewhat limited due to toxicity concerns and their unusual pharmacokinetics, highlighting the importance of developing next-generation semisynthetic glycopeptides with enhanced antibacterial activities and improved safety profiles. This Review provides an updated overview of recent advancements made in the development of novel semisynthetic glycopeptides, spanning the period from 2014 to today. A wide range of approaches are covered, encompassing innovative strategies that have delivered semisynthetic glycopeptides with potent activities against Gram-positive bacteria, including drug-resistant strains. We also address recent efforts aimed at developing targeted therapies and advances made in extending the activity of the glycopeptides toward Gram-negative organisms.

## Introduction: Antimicrobial Resistance and Glycopeptide Antibiotics

The rise of multi-drug-resistant (MDR) bacteria, paired with the decrease in the discovery of novel antibiotics, is a major threat to world health. A recent study reported that 1.27 million deaths were directly attributable to antimicrobial resistance (AMR) in 2019, with an additional 4.95 million deaths estimated to be associated with AMR.^1^ The Gram-positive pathogens methicillin-resistant *Staphylococcus aureus* (MRSA) and *Streptococcus pneumoniae* accounted for 0.5 million deaths alone in 2019.^1^ Among the therapeutic options available for treatment of such Gram-positive infections, the glycopeptide antibiotics, typified by vancomycin, have been a mainstay for many years.^2^ While the glycopeptides are among the most potent anti-Gram-positive agents available, resistance to these antibiotics is also widespread, spurring the continued search for new analogues with enhanced activities and safety profiles. From the time of its discovery, vancomycin’s structural complexity tantalized synthetic chemists, posing a monumental challenge that was ultimately met in the late 1990s, when a series of total syntheses were reported.^3−5^ In the years since, vancomycin has continued to inspire total synthesis efforts most notably aimed at generating new analogues to address and understand resistance.^6−9^ While total synthesis can provide access to glycopeptide variants otherwise unavailable in nature, semisynthesis currently presents the most practical means for accessing novel analogues in quantities suitable for clinical development. To date, a number of reviews have been published on the broad topic of the glycopeptide antibiotics.^10−17^ In this Review we provide an updated overview of recent advancements in the field, specifically as relates to the development of novel semisynthetic glycopeptides spanning the period from 2014 to today.

## Vancomycin

**Figure 1:**
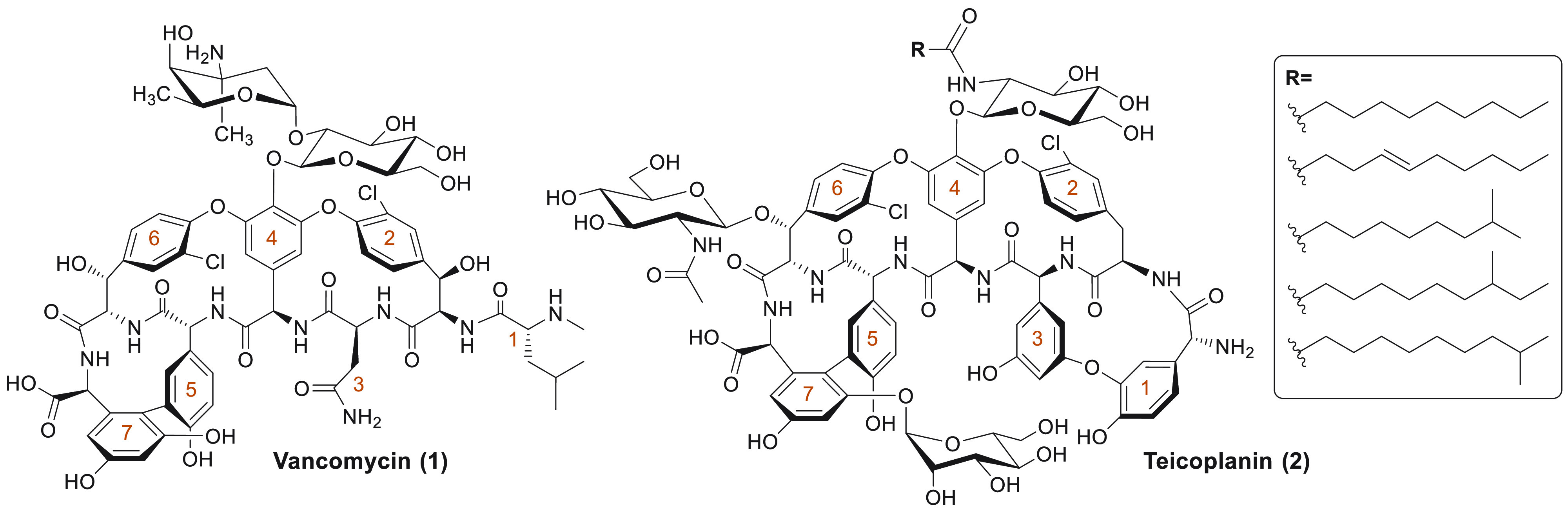
Structures of vancomycin and teicoplanin, the two clinically used natural glycopeptide antibiotics. The amino acids of the peptide are numbered in orange, starting at the N-terminus.

Vancomycin (**1**, Figure 1) was discovered in 1952, when a missionary stationed in Borneo provided E. C. Kornfield of Eli Lilly with a soil sample containing *Streptomyces orientalis*, the microorganism that produces vancomycin.^18^ Early attempts at purifying vancomycin for clinical use were challenging, leading to the nickname “Mississippi mud” due to the presence of impurities and brown color.^18^ Success in clinical trials ultimately led to the improved isolation of vancomycin, which derived its name from the word “vanquish”, given its potent antibacterial activity against a variety of Gram-positive strains, including penicillin-resistant *S. aureus*.^18^ In 1958, this novel antimicrobial agent was approved for use in the clinic.^18^ Interestingly, while aspects of vancomycin’s chemical structure were partially assigned by researchers in the 1960s and 1970s,^19−22^ it was not until 1982—some 30 years after its discovery—that a full structural elucidation was published.^23,24^ Notably, vancomycin’s clinical application was initially limited due to its less convenient intravenous (IV) route of administration, side effects, and the availability of alternative treatments such as methicillin and other β-lactams antibiotics. However, the rise of drug-resistant pathogens in the 1980s and 1990s, most notably MRSA, led to the emergence of vancomycin as the standard of care for many Gram-positive infections.^12^ The success of vancomycin subsequently led to the discovery and development of teicoplanin (**2**, Figure 1) as the only other natural product glycopeptide antibiotic to be used clinically.

**Figure 2:**
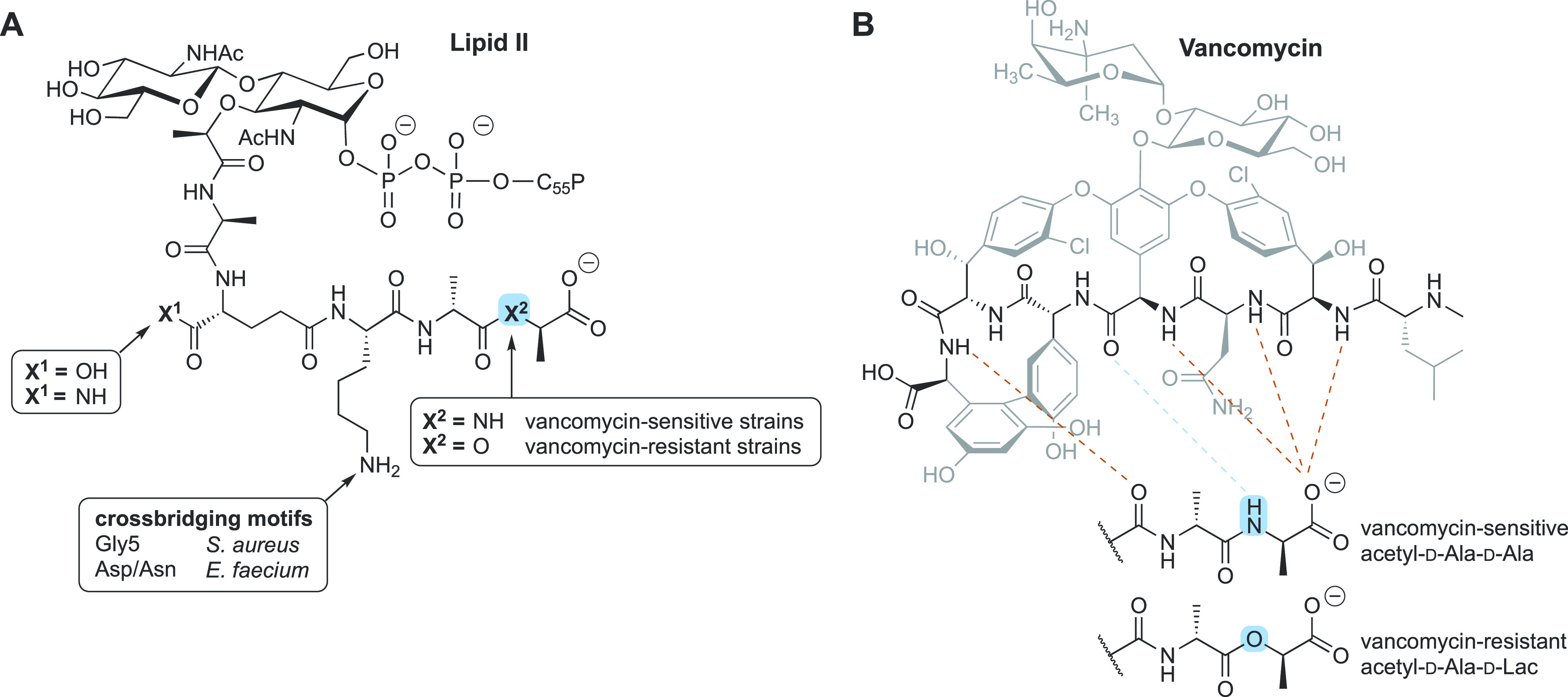
(A) Structures of lipid II found in vancomycin-sensitive and -resistant strains. Features specific to bacterial species and associated resistance are indicated. (B) Binding of vancomycin to d-Ala-d-Ala via hydrogen bonding (dotted lines). Target modification to d-Ala-d-Lac in vancomycin-resistant strains results in loss of one hydrogen bond (indicated in blue).

The antibacterial activity of vancomycin is attributable to its capacity to tightly bind the bacterial cell-wall precursor lipid II (Figure 2A) and, in turn, inhibit cell-wall biosynthesis. More specifically, vancomycin interacts with the d-Ala-d-Ala terminus of the lipid II stem pentapeptide via a well-defined network of five hydrogen bonds (Figure 2B). This interaction effectively sequesters lipid II and sterically hinders subsequent transglycosylation and transpeptidation steps, ultimately leading to the inhibition of cell-wall biosynthesis.^15,19,25−27^ The interaction of vancomycin with its target is further promoted by non-covalent cooperative self-dimerization, which serves to lower the energy barrier required to bind a second lipid II molecule on the bacterial cell surface due to co-localization.^28−30^

While the clinical use of vancomycin was accompanied by an increase in the incidence of acquired resistance to it,^2^ samples of vancomycin-resistant strains date back over 10 000 years ago, also suggesting the presence of an innate resistance reservoir.^31^ The first vancomycin-resistant enterococci (VRE) strains were reported in Europe and the U.S. in 1986 and 1987, respectively.^32−34^ Today, multiple vancomycin resistance patterns have been elucidated, with the plasmid-mediated *vanA* and *vanB* gene clusters being the predominant drivers. Expression of these resistance operons leads to target modification of the peptidoglycan precursor termini from d-Ala-d-Ala to d-Ala-d-Lac (for *vanA*, *vanB*, *vanD*, *vanF*, *vanM*) or d-Ala-d-Ser (for *vanC*, *vanE*, *vanG*, *vanL*, *vanN*).^2,12,35−38^ In the former case, the structural change leads to a >1000-fold reduction in the binding affinity of vancomycin, which can be attributed to the loss of a hydrogen bond (Figure 2B) and, more prominently, to the establishment of strong electrostatic repulsions.^39,40^ In the latter case, the effect of the d-Ser mutation is less pronounced, as it leads to only a 6-fold reduction in binding affinity.^41,42^ The *vanA* resistance operon has also been detected in *S. aureus* strains (VRSA), although it is not believed to be the main mechanism of resistance in staphylococci.^12,43,44^ Instead, the reduced vancomycin susceptibility in *S. aureus*, without the acquisition of foreign genetic material typified by vancomycin-intermediate *S. aureus* (VISA) and heteroresistant VISA (hVISA) strains, is characterized by thickened cell walls and decreased transpeptidation cross-linking activity. These phenomena lead to the accumulation of monomeric d-Ala-d-Ala-containing decoy targets, effectively hindering vancomycin in reaching the membrane surface.^12,25,45−51^

Today, vancomycin remains a first-line treatment for a variety of Gram-positive infections, including MRSA (MIC = 0.5–2 μg/mL), *S. pneumoniae* (MIC = 0.06–2 μg/mL), and *Clostridioides difficile* infections (MIC = 0.125–4 μg/mL).^2,52^ Vancomycin has been found effective in the treatment of many conditions, including endocarditis, skin and skin structure infections (SSSIs), bone infections, and airway infections.^53^ Although vancomycin can be taken orally with the purpose of reaching the colon for the treatment of *C. difficile*-associated diseases,^54^ it is preferably administered IV due to its poor oral bioavailability^55^ and the risk of VRE colonization linked to oral use.^54^ Vancomycin has a relatively low protein binding (<50%)^56−58^ and a half-life of 6–12 h in healthy adults,^56^ and is primarily eliminated unmetabolized (>80%) through renal excretion.^56,59^ Prolonged and slow infusion with vancomycin is recommended, given that one of the main toxicity concerns associated with its use is the so-called “red-man syndrome”, a histamine-mediated hypersensitivity reaction caused by mast-cell degranulation that predominantly occurs upon rapid infusion.^60−62^ Vancomycin treatment has also been linked to nephrotoxicity, particularly in patients with moderate to severe renal impairment.^63^

## Teicoplanin

Approximately 30 years after the discovery of vancomycin, the lipoglycopeptide antibiotic teicoplanin (**2**, Figure 1) was isolated from *Actinoplanes teichomyceticus*. Subsequently, teicoplanin was approved for clinical use in Europe but never for the U.S. market.^15^ Its chemical structure, elucidated in 1984,^64,65^ differs from that of vancomycin in a number of ways, including additional glycosylation sites (at positions 6 and 7), an ether-linked 4-hydroxyphenylglycine portion (position 1), and the presence of a 3,5-dihydroxyphenylglycine residue (position 3). Teicoplanin is most significantly differentiated from vancomycin by the presence of a hydrophobic acyl tail linked to the central monosaccharide moiety (at amino acid 4), which is a non-acylated disaccharide group in vancomycin.^66^ Notably, the teicoplanin fatty acid motif is actually introduced as a mixture of five related lipids, giving rise to teicoplanin A_2_-1 through A_2_-5, the ratio of which can be somewhat dictated by fermentation conditions.^67^ Generally administered as a mixture of these five similar compounds, teicoplanin has potent antibacterial activity against a variety of Gram-positive strains, including MRSA (MIC = 0.25–2 μg/mL), *S. pneumoniae* (MIC = 0.06–0.25 μg/mL), and, of particular note, VanB-type VRE (MIC = 0.25–8 μg/mL).^52,68^

Like vancomycin, teicoplanin binds the d-Ala-d-Ala motif of lipid II through a network of five hydrogen bonds^30,69,70^ but, unlike vancomycin, does not show cooperative dimerization. Any potential loss of activity due to the lack of teicoplanin self-association appears to be compensated for by the hydrophobic tail, which is hypothesized to anchor the antibiotic into the bacterial membrane, enabling localization of teicoplanin’s glycopeptide core to its lipid II target.^30,69^ While teicoplanin is generally active against VanB-type VRE strains, in which the resistance phenotype is induced exclusively by vancomycin, for VanA-type VRE and VRSA strains the resistance phenotype is also induced by teicoplanin, rendering the antibiotic inactive.^71,72^ In line with what is observed for vancomycin, reduced susceptibility to teicoplanin can also occur in a non-plasmid-mediated fashion in *S. aureus*, either as vancomycin-susceptible but teicoplanin-resistant MRSA^73^ or by displaying cross-resistance to vancomycin as in VISA/hVISA,^74^ typified by cell-wall thickening and overproduction of decoy d-Ala-d-Ala targets.^51,75^

In Europe, teicoplanin is approved for intravenous and intramuscular use in conditions caused by susceptible Gram-positive infections, including SSSIs, endocarditis, complicated urinary tract infections, bone and joint infections, pneumonia, and bacteremia.^76^ Furthermore, oral formulations are available to treat *C. difficile* infections.^76^ As opposed to vancomycin, the hydrophobic tail makes teicoplanin highly plasma protein bound (90%),^77^ and this feature is responsible for the long half-life of 100–170 h.^76^ Like vancomycin, teicoplanin is primarily excreted renally as the unchanged drug (80%).^76^ However, it is considered to have a more favorable toxicity profile compared to vancomycin, given the lower overall occurrence of adverse events, including reduced nephrotoxicity, and its limited propensity to promote histamine release.^61,78,79^

## Clinically Used Semisynthetic Lipoglycopeptide Antibiotics

**Figure 3:**
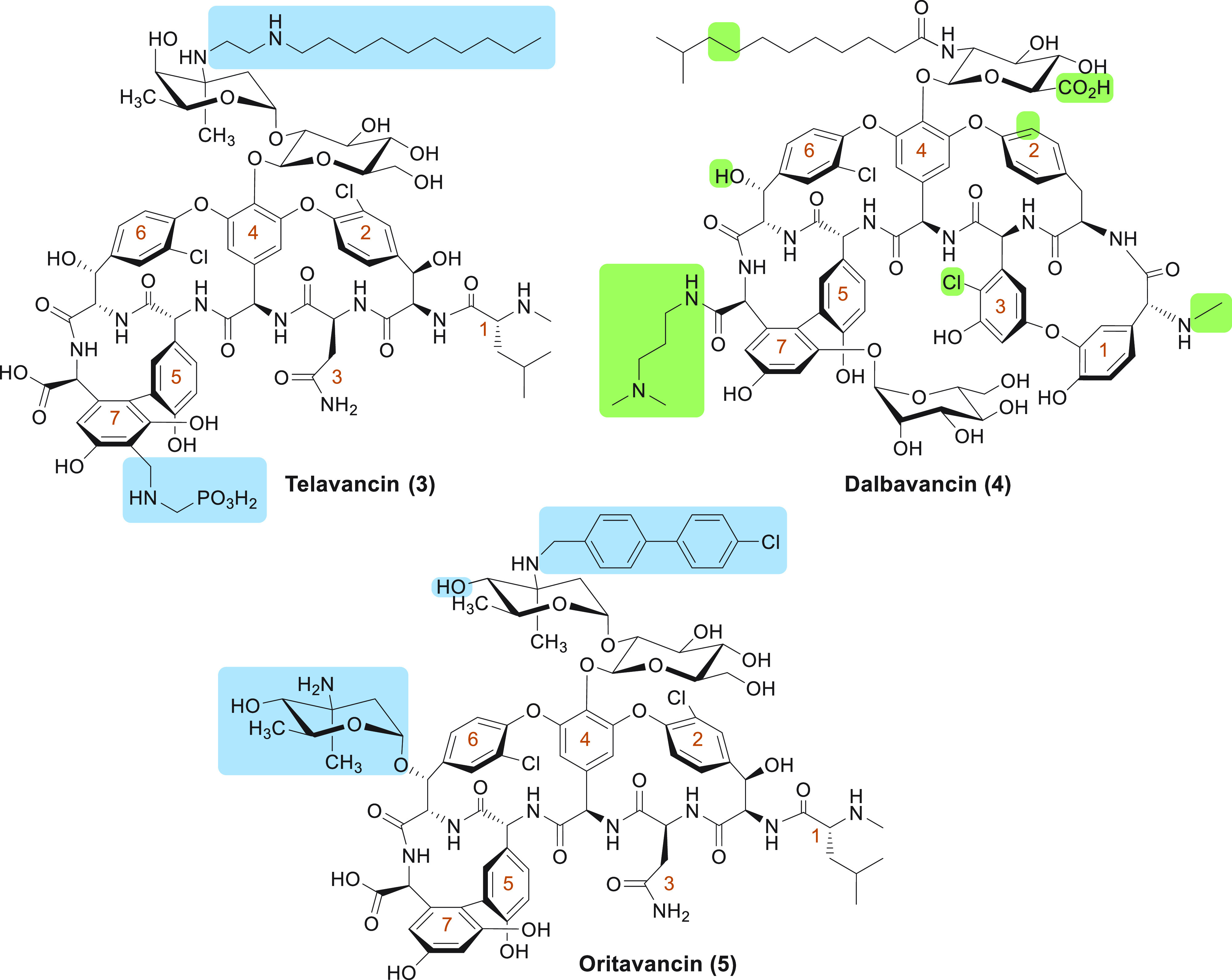
Clinically used lipoglycopeptide antibiotics. Structural differences of telavancin and oritavancin compared to vancomycin are indicated in blue. Structural differences of dalbavancin compared to teicoplanin are indicated in green. The amino acids of the peptides are numbered in orange, starting at the N-terminus.

The discovery of the natural lipoglycopeptide teicoplanin spiked interest in the development of semisynthetic lipoglycopeptide antibiotics. To date, three members of this class have been approved for clinical use: telavancin (**3**), dalbavancin (**4**), and oritavancin (**5**) (Figure 3). As noted above, a number of review articles covering the development of glycopeptide antibiotics, including telavancin, dalbavancin, and oritavancin, have been published over the years.^10−13^ However, given that these compounds present examples of successfully developed semisynthetic glycopeptide antibiotics, we will here also briefly touch upon their approval, structure, antibacterial activity, mechanism of action, resistance, clinical indications, pharmacokinetics (PK), and toxicity.

### Telavancin

Telavancin (Vibrativ, **3**, Figure 3), developed by Theravance Inc., was introduced to the clinic in 2009.^80^ It is the only clinically approved semisynthetic glycopeptide antibiotic derived from vancomycin and differs most significantly from its parent structure by the decylaminoethyl modification on the vancosamine unit, a modification that is responsible for telavancin’s enhanced potency against Gram-positive strains.^81,82^ This modification alone was found to introduce unfavorable excretion and distribution properties, and so an additional (phosphonomethyl)aminomethyl moiety was appended to ring 7, leading to an improved ADME profile.^81,82^ Telavancin is active against a variety of Gram-positive species, including MRSA (MIC = 0.016–0.125 μg/mL), VanB-type VRE (MIC = 2 μg/mL), and *S. pneumoniae* (MIC = 0.008–0.03 μg/mL).^52,83,84^ Unlike teicoplanin, it is also potent against VISA strains.^84,85^

Telavancin has a dual mode of action. First, it retains the mechanism of action of vancomycin by binding lipid II and thereby inhibiting bacterial cell wall biosynthesis.^86,87^ This interaction is promoted by the decylaminoethyl lipid, which anchors into the cytoplasmic membrane and brings telavancin into close proximity with peptidoglycan precursors. As a consequence, telavancin displays a higher binding affinity for the bacterial cell surface and increased inhibition of transglycosylation.^88^ Telavancin’s lipid moiety is also responsible for a secondary mode of action, namely the concentration-dependent dissipation of bacterial cell membrane potential (at 10-fold MIC), leading to membrane permeabilization and leakage of ATP and potassium ions.^13,86,88^ Telavancin displays a low propensity to induce spontaneous resistance in staphylococci and enterococci.^89^ Similar to teicoplanin, telavancin does not induce *vanB*, but it does effectively induce the *vanA* resistance operon.^13^ Although this leads to reduced telavancin susceptibility in VanA-type strains, this moderate increase in MIC (from ≤2 to 4–16 μg/mL)^84^ is not as drastic as the complete loss of activity seen for vancomycin and teicoplanin against these strains.^84,90,91^

Telavancin is approved to treat complicated SSSIs caused by susceptible Gram-positive species such as *S. aureus*, *Streptococcus agalactiae*, *Streptococcus pyogenes*, and *Enterococcus faecalis*.^80,85,92,93^ Furthermore, telavancin has been approved to treat hospital-acquired and ventilator-associated pneumonia when alternative treatment is not suitable.^93,94^ Due to its poor oral bioavailability, telavancin is administered IV. It is extensively plasma protein bound (93%) and has a half-life of approximately 7–9 h in healthy adults, enabling once-a-day dosing.^85,93,95,96^ Telavancin is mainly excreted through the kidneys as the intact drug (∼70%),^13^ which results in extended half-lives for patients with renal dysfunction, potentially leading to adverse effects.^97^ In relation to that, telavancin was issued a black-box warning from the FDA due to its associated nephrotoxicity concerns as well as for pregnancy-related toxicity.^93,98^

### Dalbavancin

Dalbavancin (Dalvance, **4**, Figure 3) was brought to market by Durata Therapeutics/Allergan in 2014. This semisynthetic glycopeptide is synthesized from the natural product A40926, which has a teicoplanin-like structure.^99^ However, A40926 still has significant differences in its glycopeptide core compared to teicoplanin, including the presence of a terminal methylamino group at the N-terminus (amino acid 1), the location of a chlorine atom at ring 3 rather than ring 2, decoration of residue 4 with an *N*-acylaminoglucuronic acid carbohydrate rather than with an *N*-acylglucosamine, and finally the absence of the acetylglucosamine at position 6. Furthermore, the hydrophobic acyl tail is one carbon atom longer compared to that of teicoplanin A_2_-5 (Figure 3). Dalbavancin is synthesized from A40926 by a three-step sequence, resulting in amidation of the C-terminus with 3-(dimethylamino)-1-propylamine.^100^ Dalbavancin exhibits potent activity toward Gram-positive strains, including MRSA (MIC = 0.06–1 μg/mL), streptococci (MIC ≤0.03 μg/mL), and VanB-type VRE (MIC ≤0.03–4 μg/mL).^52,101−104^

As for other glycopeptide antibiotics, dalbavancin binds to the d-Ala-d-Ala termini of cell wall precursors. While dalbavancin’s hydrophobic acyl tail may play a role similar to that found for teicoplanin in membrane anchoring and localization,^11^ the cationic dimethylaminopropyl moiety is also believed to interact with the negatively charged phospholipid head groups of the bacterial surface.^105^ Interestingly, while vancomycin dimerization is cooperative and favored upon ligand binding, dalbavancin adopts a closed conformation upon interaction with lipid II, subsequently preventing dimerization.^105,106^*In vitro* selection for resistance to dalbavancin has also been successfully demonstrated employing a *S. aureus* strain, although resistance was slower to appear than for vancomycin and teicoplanin.^107−109^ Also of note, dalbavancin-induced non-susceptible VSSA and VISA strains have also been isolated from patients; however, such accounts remain relatively uncommon.^110,111^ In line with the features of the previously discussed lipoglycopeptide antibiotics, dalbavancin is potent against VanB-type VRE strains^103^ but ineffective against VanA-type strains, as it induces the *vanA* operon.^103^ Furthermore, continuous exposure to sub-lethal dalbavancin concentrations does cause resistance selection to dalbavancin *in vitro* in VanB-type VRE over a 20-day period (MIC from 0.12 to >16 μg/mL).^112^

At present, dalbavancin is only clinically approved for the treatment of acute bacterial SSSIs,^113^ although it is increasingly used off-label for endocarditis and osteomyelitis.^114^ Similarly to other lipoglycopeptides, dalbavancin is administered IV due to its poor oral bioavailability. It has high plasma protein binding (93–98%) and displays unusual PK properties, with half-lives spanning multiple days (8.5 days),^113,115^ resulting in once-a-week dosing. Dalbavancin has a long elimination time, eventually being excreted as unaltered drug through feces (20%, 70 days) and urine (33%) or as the hydroxyl-dalbavancin metabolite through renal clearance (12%, 42 days).^113,116^ Despite its unusual PK properties, dalbavancin has an acceptable safety profile and is suited for use in patients with hepatic or mild to moderate renal impairment, with dose adjustment only required for patients with severe renal impairment.^10,116,117^

### Oritavancin

Oritavancin (Orbactiv, **5**, Figure 3) was originally developed by Eli Lilly^118^ and eventually brought to the clinic by The Medicines Company in 2014.^12^ It is derived from the naturally occurring glycopeptide chloroeremomycin and is generated semisynthetically by attachment of the 4′-chlorobiphenylmethyl group to the disaccharide moiety. Compared to vancomycin, oritavancin also bears an additional 4-epi-vancosamine monosaccharide unit attached to amino acid 6.^118^ Oritavancin has potent antibacterial activity against MRSA (MIC ≤0.008–0.5) as well as against both vancomycin-sensitive (MIC ≤0.008–0.25 μg/mL) and -resistant enterococci (MIC VanA ≤0.008–1, VanB ≤0.008–0.03).^52,119^

Besides the classical glycopeptide mechanism of action resulting from its binding to the d-Ala-d-Ala terminus of lipid II, oritavancin’s enhanced activity relative to vancomycin is ascribed to its ability to engage with secondary binding sites on lipid II. Specifically, in *S. aureus* and *Enterococcus faecium*, oritavancin is reported to also bind to the pentaglycine (Gly5) and the Asp/Asn crossbridge portion of lipid II, respectively (Figure 2B). As a result, its antibacterial activity is significantly increased and maintained even in the case of VRE strains which produce modified d-Ala-d-Lac peptidoglycan building blocks.^120−123^ Interestingly, in the case of VRSA, while the Gly5 bridge is largely absent,^124^ binding of oritavancin to the amidated α-carbonyl group of the d-glutamate residue at position 2 of lipid II appears to compensate for the loss of the key hydrogen bond associated with the d-Ala-d-Lac form of lipid II.^123^ The enhanced affinity for amidated d-Ala-d-Ala lipid II-Gly5 compared to unmodified lipid II suggests that oritavancin’s ability to target additional binding sites is responsible for its increased potency against vancomycin-sensitive strains as well.^123^ Furthermore, the tendency of oritavancin to form tight homodimers increases its affinity for the target sites.^122,125,126^ In addition to its enhanced lipid II binding, the 4′-chlorobiphenylmethyl substituent of oritavancin is thought to be involved in anchoring to the bacterial membrane, leading to localization of the antibiotic in close proximity to the membrane as well as causing dissipation of the membrane potential.^125,127−129^ Owing to its multiple modes of action, oritavancin retains activity against VRSA and VanA-type VRE, as opposed to the other clinically used glycopeptide antibiotics.^130−132^ Its multiple mechanisms of action could also lead to a lower propensity to induce resistance: while *in vitro* oritavancin resistance induction has been observed,^112,133^*in vivo* oritavancin non-susceptible strains have not been reported to date.^13,134^

Oritavancin is used clinically to treat acute bacterial SSSIs in adults caused by a variety of Gram-positive strains, including MRSA and enterococci.^135^ It is typically administered IV, displays high protein binding (>85%), and has a long half-life of 245–393 h (10.3 days), which allows for single dosing.^135,136^ Oritavancin has high tissue accumulation and prolonged retention (mainly in the liver, ≥59%), resulting in slow excretion from tissue sites, with only <5% and 1% (unmetabolized) recovery in urine and feces, respectively, after 7 days.^137^ While oritavancin generally shows low incidence of serious adverse events, when compared with a vancomycin treatment group, patients treated with oritavancin did experience higher rates of osteomyelitis as a side effect.^135,138,139^ Oritavancin is therefore not approved for the treatment of bone or bone marrow infections, and given its long terminal half-life, patients should be monitored for signs and symptoms of osteomyelitis following treatment with oritavancin.^135,138^

## Recent Developments in Semisynthetic Glycopeptide Antibiotics

### Glycopeptide Modification Sites and Chemistry

**Figure 4:**
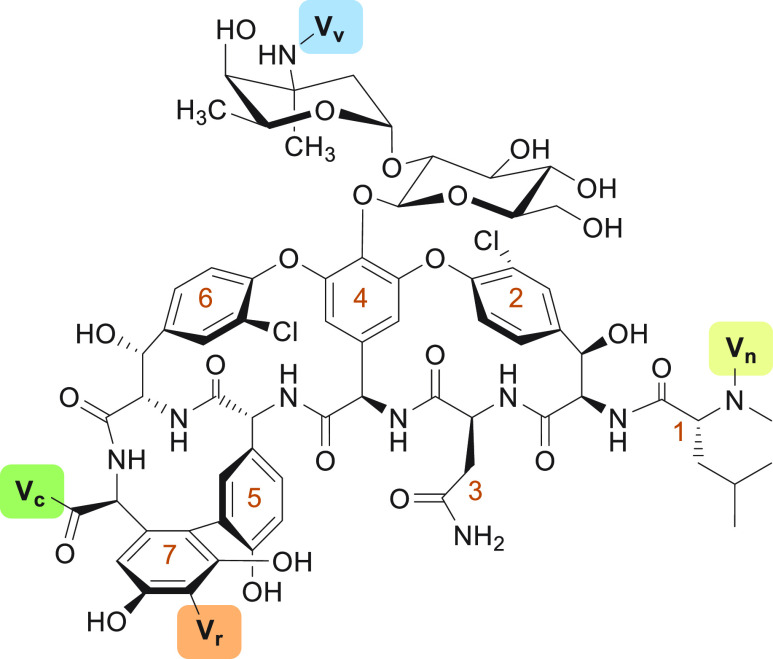
Main modification sites on vancomycin. Modifications on vancomycin are common on the vancosamine (Vv), the C-terminus (Vc), the N-terminus (Vn), and the resorcinol (Vr). The amino acids of the peptide are numbered in orange, starting at the N-terminus.

In addition to the chemical modifications associated with the clinically used semisynthetic glycopeptide antibiotics described above, many other approaches have been explored toward the development of novel semisynthetic glycopeptides. For extensive reviews on such glycopeptide derivatives, including discoveries before 2014, we refer the reader to the previous literature.^14−17^ The present Review focuses on recent advancements in the discovery of new semisynthetic glycopeptide antibiotics reported in the interval between 2014 and the present. The structural modifications made in generating novel semisynthetic glycopeptides occur largely at four defined positions: the vancosamine primary amino group (Vv), the C-terminus (Vc), the N-terminus (Vn), and the resorcinol moiety (Vr) (Figure 4). While these positions are most readily modified, structural elaboration at other sites has also been reported.^140^ The introduction of substituents at the vancosamine (Vv) motif typically relies on the selective modification of the primary amine by means of reductive amination using aldehyde-functionalized compounds. The C-terminus (Vc) is readily altered by coupling of an amine to the carboxylic acid by means of peptide bond formation. Similarly, the N-terminus (Vn) can be conjugated to carboxylic acids using strategies for making amides. Finally, the resorcinol moiety (Vr) can be functionalized using the Mannich reaction with formaldehyde and the desired amine. These four modification sites have been used to introduce a wide diversity of structural modifications aimed at (1) improving binding to the bacterial cell surface, (2) enabling multiple modes of action by adding additional binding moieties, (3) driving glycopeptide dimerization to enhance localization to the target site, (4) delivering the drug to specific target sites in the body, and (5) expanding the antibacterial spectrum of activity toward Gram-negative strains.

### Cationic (Lipo)glycopeptide Antibiotics with Enhanced Bacterial Surface Binding

**Figure 5:**
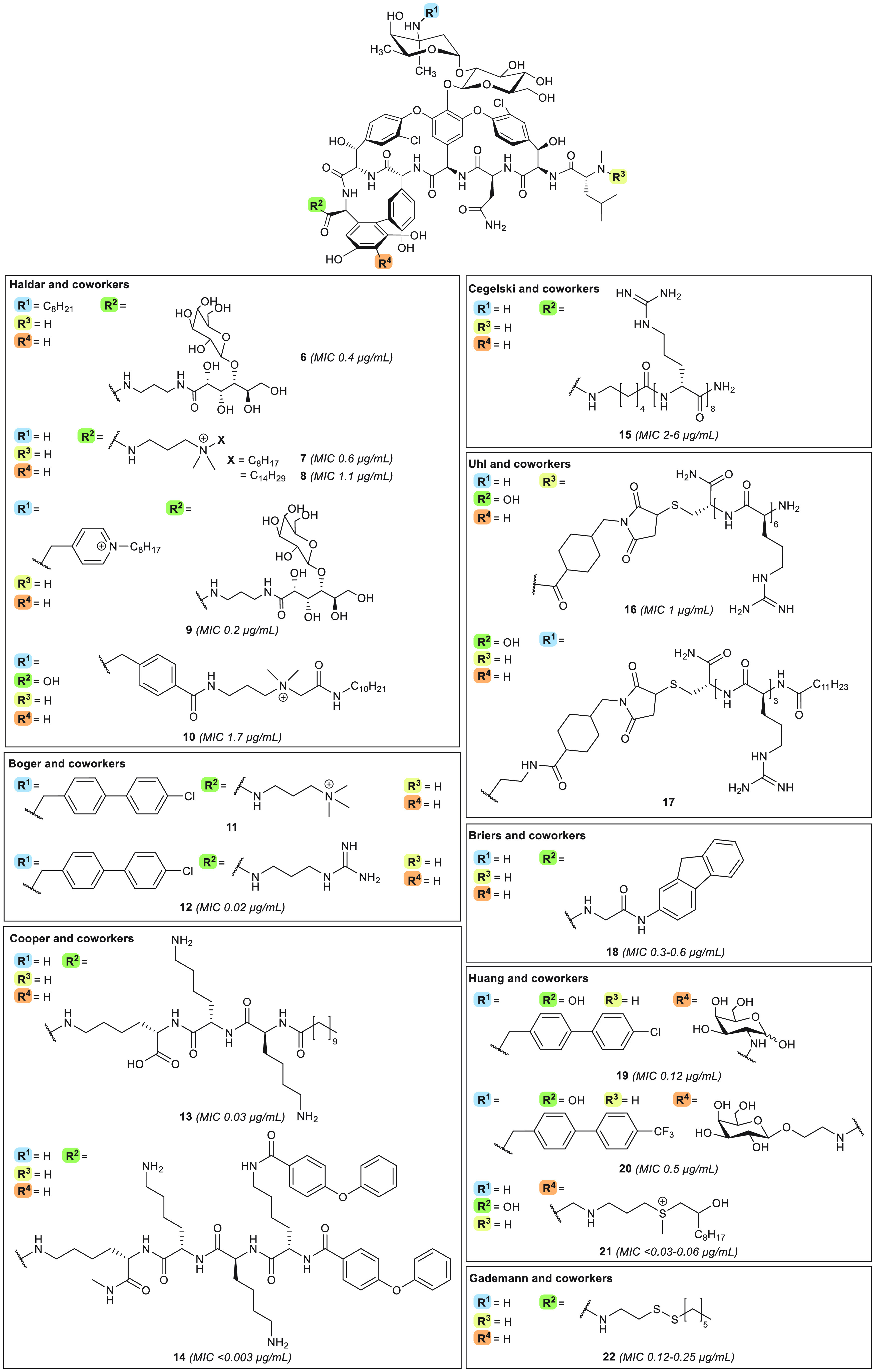
Cationic and/or lipophilic semisynthetic vancomycin analogues with enhanced cell surface binding. Compounds are organized according to research group. MIC values are indicated for MRSA strains, allowing for comparison.

Design strategies aimed at conferring semisynthetic glycopeptides with activity against vancomycin-resistant strains are usually focused on enhancing their binding to the bacterial cell surface. One of the most common approaches employed to achieve this goal is the inclusion of lipophilic substituents, as seen in the clinically used lipoglycopeptides, and/or the installation of cationic moieties that are positively charged at physiological pH, as a means of generating favorable interactions with the negatively charged bacterial cell surface. To this end, in 2014 the group of Haldar, one of the key players in the lipoglycopeptide field, appended a lipid tail to the vancosamine position and a lactobionolactone moiety to the C-terminus of vancomycin to generate compound **6** (Figure 5).^141^ Compound **6** shows potent *in vitro* activity against MRSA (MIC = 0.4 μg/mL) and VRE (MIC = 1.4–2 μg/mL) (see Table 1 for a comparative overview of the activity of the semisynthetic glycopeptides covered in this Review). Shortly thereafter, the same group conjugated two different lipophilic ammonium moieties to the C-terminus of vancomycin, yielding analogues **7** and **8** (Figure 5).^142^ Compound **8** shows potent *in vitro* bactericidal activity against MRSA (MIC = 1.1 μg/mL) and VanA-type VRE (MIC = 1.2 μg/mL) (Table 1). The enhanced potency against vancomycin-resistant strains was proposed by the authors to be due to the presence of a permanent positive charge. Subsequently, the Haldar group refined their previous findings by combining the strategies used for **6** (addition of a lipid and a carbohydrate) and compounds **7** and **8** (installation of a permanent cationic lipid), culminating in the development of the lipidated pyridinium analogue **9** (Figure 5).^143^ While inclusion of the cationic lipid alone is enough to confer excellent activity against MRSA (MIC = 0.2 μg/mL) and VRE (MIC = 4–10 μg/mL), the added carbohydrate moiety found in **9** further enhances this analogue’s potency against VanA- and VanB-type VRE strains (MIC = 0.2 and 2.7 μg/mL, respectively) (Table 1).^143^ Furthermore, **9** displays anti-MRSA-biofilm activity that leads to a 3-log titer reduction compared to vancomycin.^143^ Mechanistically, the lipophilic substituents in **6**–**9** drive the enhanced potency, while the permanent positive charges found in **7**–**9** confer membrane-disruptive properties, and the carbohydrate moiety at the C-terminus in **6** and **9** is proposed to enhance d-Ala-d-Lac binding affinity.^141−143^ Furthermore, analogues **7**–**9** show no resistance selection against MRSA.^142,143^ Given that **7** and **9** have the most favorable toxicity profiles,^142,143^ both compounds were progressed to efficacy studies, where **7** was found to exhibit a more pronounced reduction in MRSA titer in a murine thigh infection model compared to vancomycin and linezolid.^144^ In addition, **9** outperformed linezolid in a murine VRE kidney infection model by further reducing the bacterial titer 2-log.^143^ In the case of **7**, a series of further studies were aimed at evaluating its efficacy, PK, and toxicity, revealing a 50% effective dose (ED_50_) of 3.3 mg/kg and a 50% lethal dose (LD_50_) of 78 mg/kg. Moreover, compound **7** displays a prolonged half-life of 1.6 h, sustained plasma drug concentrations above MIC for at least >4 h, and no major kidney or liver damage.^144^ More recently, in 2021, Haldar and co-workers developed analogue **10**, containing a single-site vancosamine modification consisting of an aryl-ammonium-alkyl substituent, which exhibits bactericidal activity against MRSA (MIC = 1.7 μg/mL), VRSA (MIC = 0.8–3.4 μg/mL), and VRE (MIC = 0.8–6.7 μg/mL) (Figure 5, Table 1) while displaying no hemolysis or mammalian cytotoxicity.^145^ In addition to binding to d-Ala-d-Ala and delocalizing cell division proteins in cells during the exponential phase, **10** also depolarizes and permeabilizes the membrane of exponential, stationary, and persister cells. Analogue **10**, even when used at low concentrations, is able to more effectively reduce the MRSA titer and viability within biofilms compared to vancomycin.^145^ The results of these *in vitro* studies were also reflected in *in vivo* studies in mice, where **10** was found to be tolerated up to at least 55.5 mg/kg and shown to be efficacious in reducing murine MRSA thigh burden by almost 3-log compared to vehicle.^145^ Finally, analogue **10** was also found to show no resistance induction and a prolonged post-antibiotic effect.^145^

**Table 1:** *In Vitro* Antibacterial Activity against Gram-Positive Strains^a^

			MIC (μg/mL)						
category	compound		MRSA		VanA VRE		VanB VRE		refs
Clinically used	vancomycin (**1**)		0.5–2^b^		>32		>32		(52)
	teicoplanin (**2**)		0.25–2^b^		>32		0.25–8		(52, 68)
	telavancin (**3**)		0.016–0.125^b^		4–16		2		(52, 84)
	dalbavancin (**4**)		0.06–1		>32		≤0.03–4		(52, 101−104)
	oritavancin (**5**)		≤0.008–0.5		≤0.008–1		≤0.008–0.03		(52, 119)

Cationic (lipo)glycopeptide antibiotics with enhanced bacterial surface binding	**6**		0.4		1.4		2		(141)
	**7**		0.6		23.8		2.4		(142)
	**8**		1.1		1.2		nd		(142)
	**9**		0.2		0.2		2.7		(143)
	**10**		1.7		0.8–6.7^e^				(145)
	**11**		nd		0.25–0.5		nd		(146)
	**12**		0.02		0.15–0.6		0.04		(149)
	**13**, **14**		0.03, <0.003		6, 0.5		nd		(151)
	**15**		2–6		11		90		(152)
	**16**		1		≤2.7		<2.7		(153)
	**17**		nd		0.24		4.7		(154)
	**18**		0.3–0.6		1.3–21		5.2		(155)
	**19**, **20**		0.12, 0.5		2, 0.5–1		0.25, ≤0.06		(156)
	**21**		≤0.03–0.06		8		≤0.0625		(157)
	**22**		0.12–0.25		16		0.5		(158)
	**23**		0.5		0.31 to >20		0.31–1.25		(160, 161)
	**24**		0.3		0.15–2.5		0.15		(162)
	**25**		0.4		0.1–12.5		0.4		(163)
	**26**		8		8		4		(166)
	**27**		0.5		2		1		(167)
	**28**		0.125–1		≤4^e^				(169)

Pyrophosphate targeting	**29**		0.9		3.5		2.6		(173)
	**30**		4^c^		4		4		(178)

Hybrids	**31**		0.06–8^d^		8–16^e^				(185)
	**32**		1.5		6.2		nd		(188)
	**33**		0.6		nd		0.8		(190)
	**34**		6.25–12.5		12.5–25^e^				(191)
	**35**		4		4		8		(192)
	**36**		4		8		4		(192)

Targeted drug delivery	**37**		0.79^f^		28.9^g^		28.9^g^		(200)
	**38**		2		nd		nd		(202)
	**39**		nd		nd		nd		(205)
	**40**		0.015		0.03–2		0.03		(210)

Gram-negative active	**8**		1.1		1.2		nd		(142, 211)
	**41**		0.7		3.8		6.9		(212)
	**42**		15–30		nd		nd		(213)
	**43**		8^c^		32		nd		(214)
	**44**		0.25		64 to >128		2–64		(215)
	**45**		0.8^d^		nd		nd		(216)
	**46**		0.5		nd		nd		(217)
	**47**		nd		nd		nd		(218)
	**48**		4^d^		nd		nd		(219)

a MIC = minimum inhibitory concentration. nd = not determined.

b MIC values of >10 observations are included in the reported MIC range from EUCAST.^52^

c MRSA strain tested was also VISA.

d No MRSA strain was tested; therefore, an MIC range for MSSA is indicated here.

e MIC reported is from VRE in general, as literature did not specify VanA- or VanB-type resistance. Note the possibility of solely one *van* resistance type being present.

f Low-density loading of nanoparticles (0.2 μg/mL vancomycin per 1 mg of **37**).

g High-density loading of nanoparticles (11.75 μg/mL vancomycin per 1 mg of **37**).

In 2017, Boger and co-workers appended the 4′-chlorobiphenylmethyl (CBP) unit, also found in oritavancin, to the vancosamine site of vancomycin and added a quaternary ammonium at the C-terminus (Figure 5). These modifications resulted in compound **11**, which was found to display *in vitro* antibacterial activity against VanA-type VRE (MIC = 0.25–0.5 μg/mL) (Table 1).^146^ Analogue **11** also binds the d-Ala-d-Ala motif of lipid II, inhibits cell wall biosynthesis via direct competitive inhibition of transglycosylases (owing to the CBP motif), rapidly permeabilizes and depolarizes the bacterial cell membrane (by virtue of the trimethylammonium portion), and binds to teichoic acids (due to the trimethylammonium moiety).^146−148^ In a follow-up publication, the same group further optimized compound **11** by retaining the CBP unit but replacing the trimethylammonium group with a guanidine moiety, hypothesized to serve as a beneficial hydrogen bond donor, to yield analogue **12**.^149^ Analogue **12** was found to display *in vitro* potency against MRSA (MIC = 0.02 μg/mL), VanA-type VRE (MIC = 0.15–0.6 μg/mL), and VanB-type VRE (MIC = 0.04 μg/mL) (Figure 5, Table 1). Mechanistically, compounds **11** and **12** are comparable^149^ and share the key feature of a positively charged substituent (at physiological pH) situated at the vancomycin C-terminus. The importance of this structural trait is demonstrated by the fact that relocating motifs of a cationic nature elsewhere on the antibiotic core does not enhance potency and only slightly alters the initial rate of membrane permeabilization.^147,149,150^ While both analogues showed no mammalian cytotoxicity^146,148^ and exhibited good *in vivo* tolerability (≥50 mg/kg in mice),^146,149^ compound **12** appears superior to **11** by virtue of having (1) a lower propensity to induce resistance against VRE (>10-fold MIC increase for **11**, marginal changes for **12**)^148,149^ and (2) superior *in vivo* efficacy in a murine VRSA thigh infection model at 12.5 mg/kg (4-log versus 5-log reduction for **11** and **12**, respectively, when compared to vancomycin).^148,149^ The half-lives of **11** and **12** in mice are 6–7 and 4.3 h, respectively.

Also with an eye to introducing cationic and lipophilic features onto the vancomycin core, Blaskovich and Cooper designed the vancaptins.^151^ The vancaptins feature an additional C-terminal peptide, bearing numerous positively charged functionalities, followed by a lipophilic membrane-insertive element, and are represented by compounds **13** and **14** (Figure 5). Against MRSA, the vancaptins were found to be 20- to 100-fold more active than vancomycin and daptomycin (MIC **13** and **14** < 0.003–0.03 μg/mL) (Table 1), along with having enhanced potencies against VISA (0.125–0.5 μg/mL), VRSA (0.08–1 μg/mL), *S. pneumoniae* (<0.003–0.06 μg/mL), and VanA-type VRE (0.5–6 μg/mL).^151^ These *in vitro* data were also found to correlate well with the *in vivo* activity of the vancaptins, where treatment with **13** and **14** led to 100% survival in a *S. pneumoniae* murine infection model. Furthermore, **13** was shown to effectively reduce murine MRSA thigh burden by 6-log compared to vehicle when employing a dose 8 times lower than that required of vancomycin to gain the same effect. Interestingly, compound **14** was found to be less effective *in vivo*, which was ascribed to its high protein binding, given that PK studies indicated that both **13** and **14** reach an *in vivo* concentration above their MIC values for more than 8 h. Additionally, the vancaptins were shown to be bactericidal, non-hemolytic, and non-toxic to mammalian cells (CC_50_ ≥ 100 μM) and to cause minimal resistance induction in MRSA. Mechanistic studies further revealed that the vancaptins exert their antibiotic effect through multiple modes of action by (1) inhibiting cell-wall biosynthesis by binding to d-Ala-d-Ala, (2) increasing membrane binding and cooperative dimerization similar to vancomycin, and (3) depolarizing and perturbing the cell membrane (most prominently in the case of compound **14**).^151^

While the strategies described above mainly focused on appending cationic and lipophilic substituents to vancomycin, other groups have opted to focus solely on the introduction of additional positive charges, leading to conjugation of polyarginine motifs to vancomycin as in analogues **15**(152) and **16**(153) (Figure 5). To this end, the groups of Wender and Cegelski generated **15**, modified at the C-terminal position with an octaarginine peptide, which was found to exhibit good potency against MRSA (MIC = 2–6 μg/mL).^152^ Using a similar approach, Uhl and co-workers examined the effect of introducing a hexaarginine moiety at the four different sites of vancomycin indicated in Figure 4.^153^ This led to identification of the N-terminally modified **16** as the most potent variant, with good activity against MRSA (MIC = 1 μg/mL) and VRE (MIC ≤2.7 μg/mL) (Table 1).^153^ Interestingly, the activity of **16** is not antagonized by d-Ala-d-Ala, suggesting that an alternative mode of action is responsible for the enhanced potency of this derivative.^153^ The mechanism of action of the hexaarginine-substituted compound is likely similar to that of analogue **15**, for which enhanced binding to the membrane, driven by strong electrostatic interactions, facilitates cellular association, along with internalization to give access to intracellular peptidoglycan precursors.^152^ Additionally, these compounds also display rapid membrane permeabilization, although only during cell growth.^152^ Both **15** and **16** are active *in vivo*, with **16** reducing murine MRSA thigh burden similarly to vancomycin.^153^ Compound **15** was found to display a 6-fold potency enhancement in a murine MRSA biofilm wound model when compared with a similar dose of vancomycin.^152^ The *in vivo* anti-biofilm activity of **15** was also demonstrated with *in vitro* experiments, wherein treatment of pre-formed MRSA biofilms with **15** resulted in significantly reduced cell viability to 8.4% after 5 h, compared to 65% viability for vancomycin-treated biofilms. Furthermore, the unique ability of **15** to target biofilms was demonstrated by the finding that combinations of vancomycin with an octaarginine peptide failed to show any anti-biofilm activity.^152^ Building upon their findings with compound **16**, Uhl and co-workers also examined the impact of adding lipophilic moieties by conjugating lipidated triarginine motifs at three different sites on vancomycin (Vv, Vc, Vn). From this series of analogues, vancosamine-modified **17** was found to be the most potent derivative, with MIC = 0.24–4.7 μg/mL against VRE (Figure 5, Table 1). This result is in stark contrast with the finding that, when appending a hexaarginine moiety, the best antibiotic activity was seen for compound **16**, modified at the N-terminus.^154^ Both **16** and **17** are non-hemolytic and non-toxic toward liver and kidney cells. Moreover, *in vivo* mice experiments with **16** and **17** revealed that the compounds reside in the liver for several hours and do not primarily distribute to the kidneys, unlike vancomycin,^153,154^ a behavior which could alleviate the risk of nephrotoxicity in patients with renal impairment.^63^

The design of vancomycin derivatives that focus exclusively on the incorporation of lipophilic moieties has also been explored, resulting, for example, in fluorenyl-substituted compound **18** reported by Briers and co-workers in 2018 (Figure 5).^155^ Analogue **18** is bactericidal against MRSA (MIC = 0.3–0.6 μg/mL) and bacteriostatic against VanA-type VRE (MIC = 1.3–21 μg/mL) and VanB-type VRE (MIC = 5.2 μg/mL) (Table 1), while displaying low toxicity against mammalian cell lines (CC_50_ = 172 μM) and minimal resistance selection against VRE.^155^ In the same year, the Huang group investigated the effect of attaching additional carbohydrate moieties onto lipophilic vancomycin analogues, culminating in compounds **19** and **20**, both bearing a carbohydrate substituent at the resorcinol position along with hydrophobic *p*-Cl- or *p*-CF_3_-biphenylmethyl moieties attached at the vancosamine site (Figure 5).^156^ Both **19** and **20** exhibit strong *in vitro* activity against MRSA (MIC = 0.12 and 0.5 μg/mL, respectively), VanA-type VRE (MIC = 2 and 0.5–1 μg/mL respectively), and VanB-type VRE (MIC = 0.25 and ≤0.06 μg/mL, respectively) (Table 1). When evaluated in an *in vivo* murine MRSA survival study, **19** and **20** respectively led to a 14/15 and 13/15 survival after 10 days as well as a >1-log reduction of liver colony-forming units compared to vehicle and vancomycin in a VISA abscess formation assay.^156^ The *in vivo* PK properties of compounds **19** and **20** were also assessed, revealing prolonged half-lives (∼3–4 h), with retained plasma concentrations of >1 μg/mL for 4 h. These studies also showed that incorporation of the carbohydrate moiety at the resorcinol position can be used to attenuate the compound’s half-life.^156^ Mechanistic studies employing NMR and molecular modeling indicate that the added carbohydrate motif might also contribute to antibacterial activity by interaction with d-Ala-d-Ala,^156^ a finding in line with the enhanced target binding Haldar and co-workers also reported for their carbohydrate-modified analogues **6** and **9**.^141,143^

The Huang group also explored the addition of cationic functionalities to vancomycin, but instead of the commonly employed ammonium or guanidinium moieties, they assessed the effect of adding sulfonium groups.^157^ The series’ lead compound **21** (Figure 5), consisting of a resorcinol-linked alkyl-sulfonium moiety, was shown to have potent activity against MRSA (MIC ≤0.03–0.06 μg/mL) and VanB-type VRE (≤0.0625) as well as moderate MIC reductions relative to vancomycin against VanA-type VRE (to 8 μg/mL) and *Escherichia coli* (to 32 μg/mL) (Table 1). Murine MRSA and VRSA infection survival studies found that treatment with **21** led to 13/15 and 12/15 survival, respectively, at 14 days, a significant improvement compared to vancomycin (3/15 survival). To investigate the specific impact of the sulfonium group on PK and toxicity, compound **21** was compared to the corresponding thioether analogue. This showed that **21** has a shorter half-life (1.13 h), an unchanged MIC in the presence of human serum albumin, and less of an effect on mammalian cell viability relative to the thioether.^157^ The authors hypothesized that analogue **21** interacts with the negatively charged bacterial membrane via the sulfonium motif, subsequently facilitating permeabilization by means of the lipophilic tail. As the thioether-linked compound does not show membrane permeabilization, it can be concluded that the charged sulfonium portion is essential to enable this mechanism of action.^157^

Gademann and colleagues also designed sulfur-modified vancomycin derivatives, but these do not comprise positively charged substituents.^158^ Compound **22** (Figure 5), bearing a disulfide-linked lipid at the C-terminal position, was found to possess potent activity against MRSA (MIC = 0.12–0.25 μg/mL), *S. pneumoniae* (MIC = 0.06 μg/mL), and VanB-type VRE (0.5 μg/mL) (Table 1). Furthermore, **22** was also shown to suppress MRSA and VRE biofilm formation (MBIC = 1 and 2 μg/mL, respectively).^158^ Given these positive results, it would be interesting to study the influence of the disulfide on PK and toxicity relative to that of the all-carbon-based compound: the potential reductive lability of **22** might be expected to lead to decomposition *in vivo* to generate more hydrophilic metabolites, thereby reducing tissue accumulation and promoting excretion, as previously noted by researchers at Theravance Inc. working with similar vancomycin analogues.^159^

**Figure 6:**
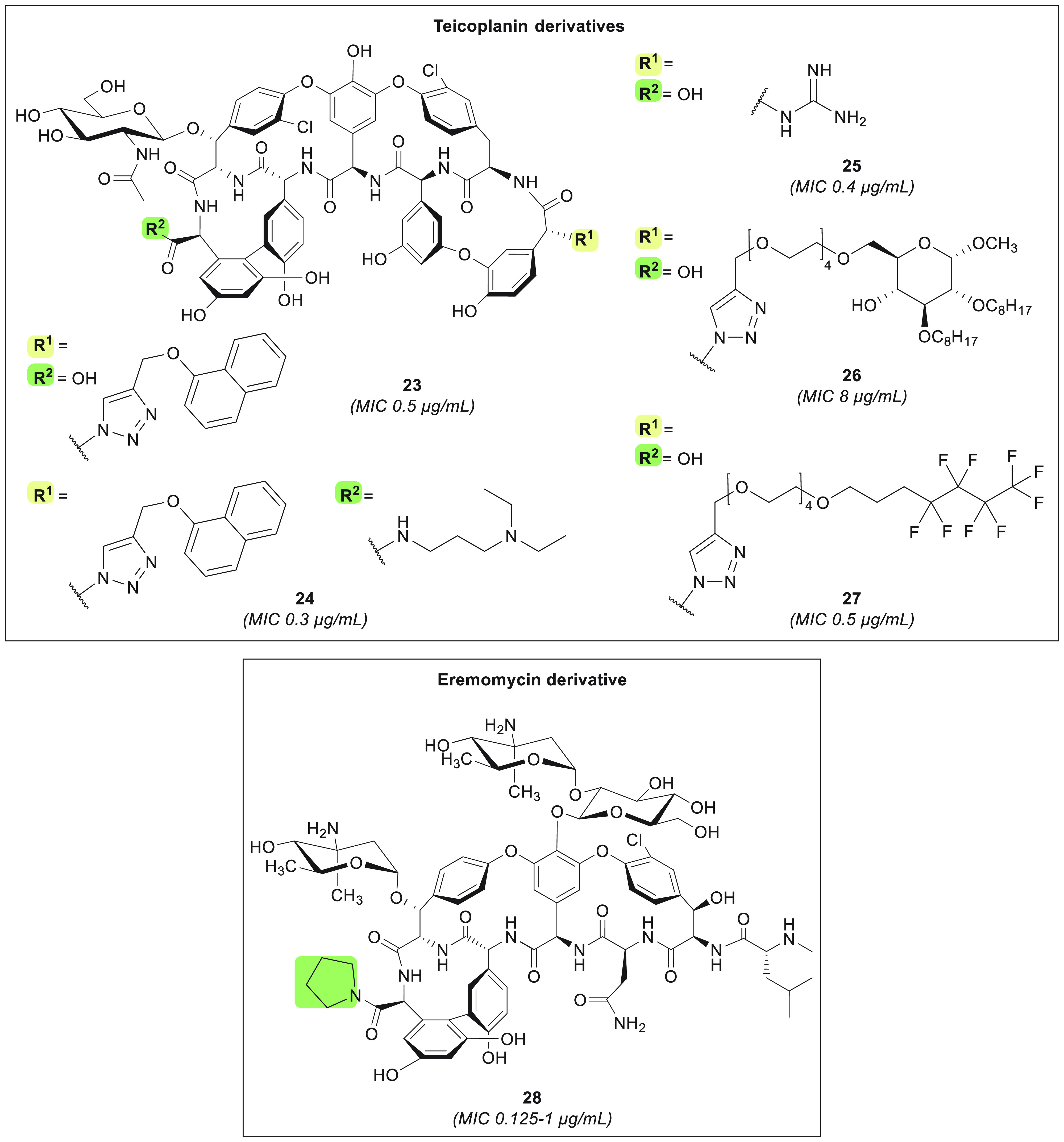
Teicoplanin and eremomycin derivatives with enhanced cell surface binding. MIC values are indicated for MRSA strains, allowing for comparison.

In addition to semisynthetic analogues of vancomycin, derivatives of teicoplanin and eremomycin have also been explored in recent years. Herczegh and co-workers designed a series of teicoplanin pseudo-aglycon compounds featuring N-terminal conjugation with various hydrophobic substituents which were introduced through azide–alkyne cycloaddition (Figure 6).^160,161^ Among the analogues thus prepared, compound **23** was found to have good activity against MRSA (MIC = 0.5 μg/mL) and VanB-type VRE (MIC = 0.31–1.25 μg/mL) (Table 1). Furthermore, some but not all VanA-type VRE isolates were found to be susceptible to this novel teicoplanin derivative (MIC = 0.31 to >20 μg/mL), as well as some strains carrying both *vanA* and *vanB* (MIC = 1.25 to >20 μg/mL).^161^ Optimization of **23** led to compound **24**, characterized by the addition of a basic moiety at the C-terminus, which displayed improved activity against VanA-type VRE (MIC = 0.15–2.5 μg/mL) while retaining potency against MRSA (MIC = 0.3 μg/mL) and VanB-type VRE (MIC = 0.15 μg/mL) (Table 1).^162^ In another attempt to confer anti-VanA-type VRE activity to teicoplanin-like compounds, analogue **25**, bearing an N-terminal guanidine moiety, was also synthesized.^163^ This led to a vast improvement in potency toward *vanA* VRE isolates, with most strains tested showing susceptibility (MIC = 0.1–1.6 μg/mL) and with only a few strains exhibiting higher MIC values (6.25–12.5 μg/mL). The ability of compound **25** to engage in additional hydrogen bonding via the guanidine moiety is assumed to contribute to the enhanced activity, although experimental evidence in support of this claim is yet to be reported.^163^ Interestingly, analogue **23** was also found to possess antiviral activity against several influenza strains,^160^ leading Herczegh and colleagues to design teicoplanin derivatives with structural features aimed at potentiating their antiviral action.^164−168^ Some of these compounds, modified at the N-terminus with lipophilic moieties linked through a triazole, still retain some antibacterial activity (see compounds **26** and **27**) (Figure 6, Table 1).^166,167^ Of these dual antibacterial and antiviral derivatives, compound **27** displays the most favorable toxicity profile (CC_50_ = 97–100 μM)^160,166,167^ while maintaining potent antibacterial activity against MRSA (MIC = 0.5 μg/mL) and VRE (MIC = 1–2 μg/mL).^167^

In a study involving the preparation of semisynthetic eremomycin analogues, Olsufyeva et al. showed that coupling small substituents to the C-terminus can be sufficient to enhance potency (Figure 6).^169^ Using this approach, they identified eremomycin pyrrolidide analogue **28**, which was found to exhibit good *in vitro* activity against MRSA (MIC = 0.125–1 μg/mL) and VRE (MIC ≤4 μg/mL) (Table 1) along with *in vivo* activity against *S. aureus* (ED_50_ = 0.8 mg/kg, 100% survival at 2.5 mg/kg). Moreover, analogue **28** was shown to be superior to vancomycin and eremomycin in a murine sepsis model, maintaining similar *in vivo* acute toxicity but eliciting reduced histamine release.^169^

As illustrated in the preceding section, a number of the recently reported semisynthetic glycopeptides exhibit enhanced activity that is associated with an increase in net positive charge most commonly achieved by incorporation of (1) permanently positively charged substituents (e.g., tertaalkylammonium, sulfonium) and/or (2) functional groups that are positively charged at physiological pH (e.g., amine, guanidine). While many of these compounds show promising *in vitro* and, in some cases, *in vivo* potency, special attention should be paid to their toxicity and PK profiles. Another structural modification commonly associated with improved antibacterial potency is the introduction of lipophilic substituents that confer these semisynthetic glycopeptides with membrane depolarizing and permeabilizing properties. However, this can also lead to enhanced toxicity and unusual PK behavior. That said, it is possible that such issues can be addressed by structure–relationship activity studies to establish optimal lipid lengths or by the use of reductively labile disulfide-linked lipids. In addition, the introduction of hydrophilic moieties, such as carbohydrates, also provides a means for fine-tuning the PK properties of semisynthetic glycopeptides.

### Pyrophosphate-Targeting Glycopeptides

**Figure 7:**
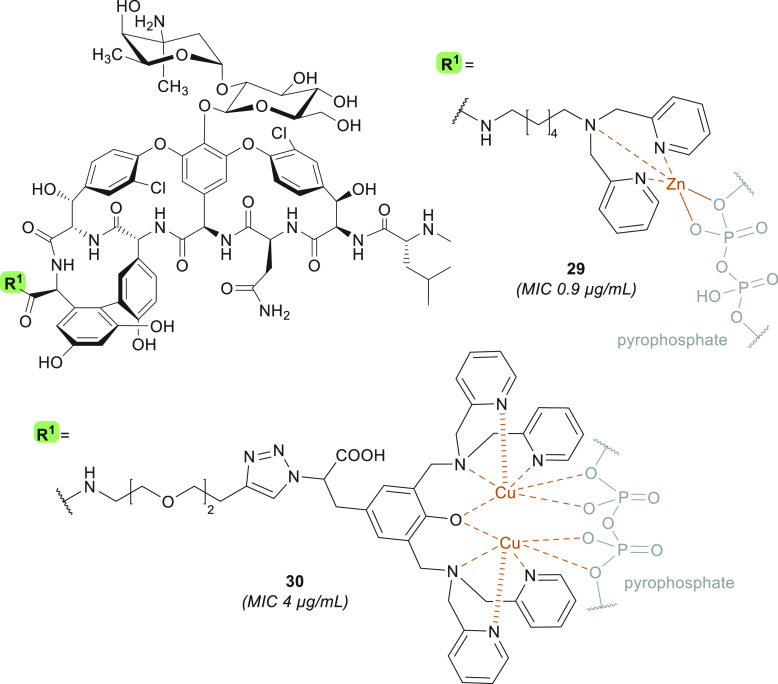
Pyrophosphate-targeting glycopeptides **29** and **30**. Derivative **30** was assessed as a Cu^2+^ chelation complex as well as a non-metal DPA analogue, in both cases displaying equipotent *in vitro* activity. MIC values are relative to experiments carried out on MRSA strains.

As demonstrated by oritavancin, the design of glycopeptide antibiotics capable of binding to lipid II at multiple sites is a viable strategy for enhancing antibacterial activity: this approach can increase potency against vancomycin-sensitive strains as well as compensate for the loss in binding affinity to the d-Ala-d-Lac motif in vancomycin-resistant strains. One such additional binding site explored in this regard is the pyrophosphate moiety of lipid II, a target that is exploited by natural product antibiotics such as nisin, ramoplanin, and teixobactin.^170−172^ To this end, Haldar and co-workers reported the design of Dipi-van (**29**) (Figure 7). Compound **29** bears a C-terminal zinc-binding dipicolyl-1,6-hexadiamine moiety,^173^ a functionality known to have a high affinity for pyrophosphates.^174^ Compound **29** was found to exhibit potent activity against VISA as well as VanA-type and VanB-type VRE (MIC = 1.8–3.5 μg/mL) (Table 1),^173^ an effect that was shown to be further enhanced some 2- to 3-fold by the exogenous addition of Zn^2+^.^173^ The expected dual mode of action, based on binding to both the pyrophosphate and the d-Ala-d-Ala motifs of lipid II, was confirmed.^173^ Analogue **29** displays no resistance selection in MRSA (MIC remained ∼0.9 μg/mL), no hemolytic activity or mammalian cytotoxicity (at 1 mM), and no systemic *in vivo* toxicity (at 100 mg/kg).^173,175^ Furthermore, in a murine renal VanB-type VRE infection model, **29** (dosed at 12 mg/kg) reduces the bacterial titer up to 5-log compared to vehicle and 3-log compared to the same dose of vancomycin.^173^ Interestingly, the Zn^2+^-binding properties of **29** not only enhance its potency against Gram-positive species but also resensitize several NDM-1-producing Gram-negative strains to meropenem by removing the zinc ions bound to the metallo-β-lactamase, a well-documented mode of action exploited by anti-NDM antibiotic potentiators such as aspergillomarasmine A^176^ and dipicolinic acid derivatives.^177^ In this regard, co-administration of vancomycin derivative **29** with meropenem was found to cause a reduction in the MIC of meropenem from >100 to 1.5–3.1 μg/mL in *Klebsiella pneumoniae* and 12 μg/mL in *E. coli* (FIC ≤0.5).^175^ This *in vitro* synergy was also further substantiated *in vivo*, specifically in a sepsis model of an NDM-positive *K. pneumoniae* infection, where a combination treatment of meropenem and compound **29** reduces the bacterial load by 3–4 log compared to vehicle in the liver, kidneys, spleen, and lungs of mice. These results are on par with those obtained with colistin treatment but superior to those gathered using **29** or meropenem monotherapy, which resulted in a maximum 1.5-log reduction in the organs assessed.^175^

Huang and co-workers also explored the possibility of developing semisynthetic glycopeptides capable of targeting the pyrophosphate group of lipid II by conjugating Cu^2+^-dipicolylamine (DPA) complexes to either the resorcinol position or the C-terminus of vancomycin.^178^ Representative compound **30** (Figure 7) was shown to have enhanced activity against VRE strains (MIC = 4 μg/mL) but not against MSSA and VISA (Table 1).^178^ A dye displacement assay confirmed that both Cu(II)- and Zn(II)-**30** complexes bind to pyrophosphoric acid, suggesting a dual mechanism of action wherein the decreased affinity for d-Ala-d-Lac is compensated for by pyrophosphate binding. Interestingly, the copper-containing **30** and the corresponding metal-free ligand are equipotent *in vitro*, but the presence of copper results in reduced cell viability (at >50 μM), suggesting that the latter DPA derivative shows more promise.^178^ Overall, pyrophosphate-targeting glycopeptide derivatives **29** and **30** display significant improvements in VanA-type VRE activity, while maintaining potency against other Gram-positive species.

### Glycopeptide Hybrid Antibiotics

**Figure 8:**
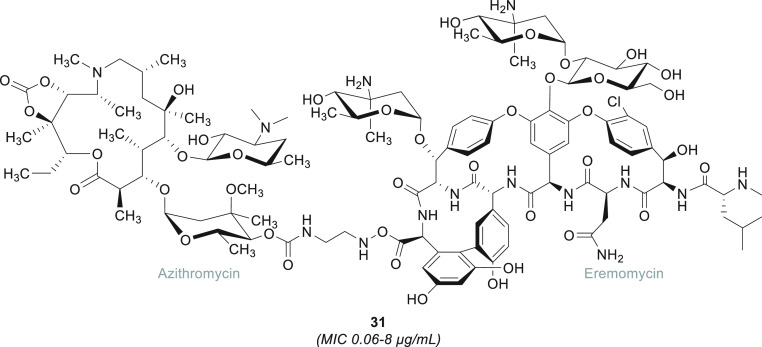
Glycopeptide–azithromycin hybrid. The eremomycin–azithromycin hybrid **31** is the most potent representative of a panel of glycopeptide–azithromycin analogues designed by Batta and co-workers.^185^ MIC values are relative to experiments carried out on MSSA strains.

Another strategy often explored to achieve antibiotics with a dual mode of action is based on the design of hybrids wherein two different antibiotic molecules are covalently linked together. A suggested benefit of this approach is the reduced likelihood of resistance induction, which is minimized by the inherent difficulties in simultaneously mutating multiple targets.^179^ Earlier strategies in this field resorted to conjugating glycopeptides to β-lactam antibiotics^180−182^ or antimicrobial peptides such as nisin(1–12) and tridecaptin.^183,184^ More recently, the group of Batta and co-workers reported the development of glycopeptide–azithromycin hybrids (Figure 8).^185^ Coupling azithromycin, a macrolide antibiotic that inhibits the assembly of the 50S ribosomal subunit used to treat Gram-positive infections,^186^ to the C-terminus of eremomycin resulted in derivative **31**, which displays *in vitro* activity against *S. aureus* and *S. pneumoniae* (MIC = 0.06–8 μg/mL) and moderate potency against VRE (MIC = 8–16 μg/mL).^185^ Compound **31** retains the mechanism of action of the azithromycin fragment and, in an *in vitro* setting, is 4-fold more potent than vancomycin against *S. aureus*. During *in vivo* experiments in a murine sepsis model with the same strain, hybrid **31** was shown to be equipotent to vancomycin, with both having an ED_50_ of 4 mg/kg.^185^

**Figure 9:**
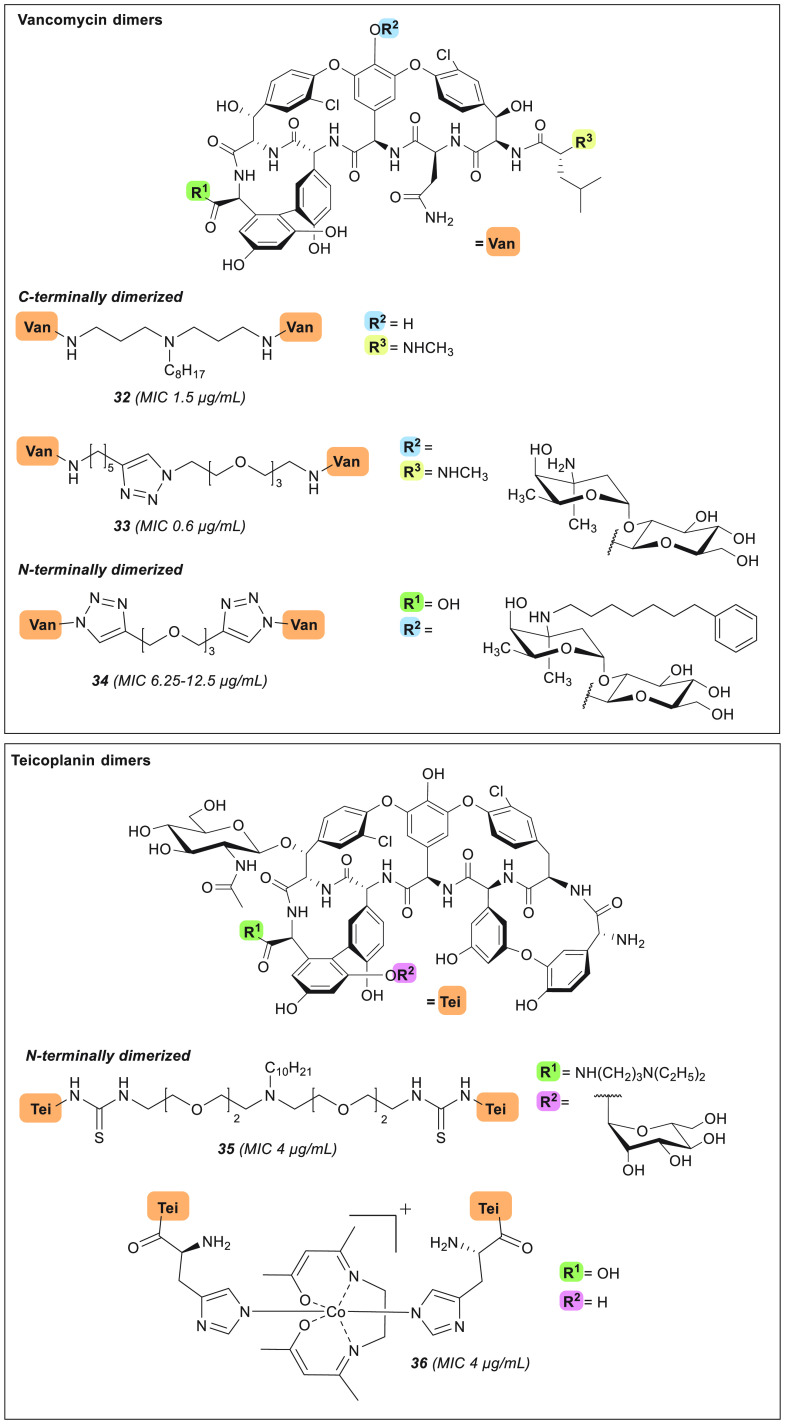
Glycopeptide dimers. MIC values are relative to experiments carried out on MRSA strains.

In addition to the hybridization of glycopeptides with other antibiotics endowed with a complementary mode of action, covalent homodimerization is another strategy for improving antibacterial potency. An exemplary example of this behavior is inspired by vancomycin, which cooperatively self-associates to form non-covalent dimers as part of its inherent mode of action. The presence of dimers leads to co-localization of the glycopeptide to its target site and reduces the energy required for a second binding event to lipid II, which results in an improved antimicrobial activity.^29,30^ The fact that this self-association occurs only weakly (700 M^–1^) in solution^187^ prompted the scientific community to explore the covalent dimerization of vancomycin, of which the first examples were reported in 1996 by Griffin and colleagues.^187^ More recently, Haldar and co-workers revisited this approach by synthesizing a number of bis(vancomycin aglycon)carboxamides, which are composed by homodimers of vancomycin aglycon linked through the C-terminus by lipophilic cationic spacers.^188^ One of the members of this series, compound **32**, was found to retain activity against MRSA (MIC = 1–1.5 μg/mL) and displayed a 300-fold enhanced potency against VRE (MIC = 6.2 μg/mL) compared to vancomycin (Figure 9, Table 1).^188^ The binding affinity of **32** for *N*,*N*′-diacetyl-Lys-d-Ala-d-Ala was demonstrated to be similar to that of vancomycin, while notably a >10-fold enhancement toward *N*,*N*′-diacetyl-Lys-d-Ala-d-Lac was also measured.^188^ Interestingly, this result is in stark contrast to the absence of d-Ala-d-Lac binding displayed by previously studied vancomycin dimers, as reported by Ellman and co-workers.^189^ Further assessment of the activity of dimer **32** in an *ex vivo* whole blood study showed that **32** (dosed at 2 μM) causes a 1.5-log reduction of bacterial MRSA titer in comparison to vancomycin (dosed at 4 μM), suggesting that antibacterial activity is not significantly impacted by binding to plasma proteins. These results were also in line with the different *in vitro* killing kinetics the Haldar group observed wherein compound **32** was found to be bactericidal while vancomycin functions as bacteriostatic against higher-inoculum stationary phase MRSA.^188^

Another convenient approach for generating vancomycin dimers is through the use of the copper-catalyzed azido-alkyne cycloaddition (CuAAC), as applied by the group of Sharpless, who prepared a panel of vancomycin homo- and heterodimers characterized by different alkyl and PEG spacers (Figure 9).^190^ The heterodimers, constructed by linking the C-terminus (Vc) of one vancomycin unit to the vancosamine (Vv) moiety of the other, showed no enhanced potency relative to vancomycin itself. However, in the case of the homodimers prepared, improved activity was observed, with the most potent C-terminal homodimer **33** exhibiting strong *in vitro* activity against MRSA (MIC = 0.6 μg/mL) compared to vancomycin (MIC = 2.5 μg/mL) (Table 1).^190^ In addition, **33** is >30-fold more active than vancomycin against a VanB-type VRE strain (MIC = 0.8 μg/mL).^190^ In a similar study, Sun and colleagues also utilized CuAAC chemistry to obtain covalent glycopeptide dimers typified by compound **34** (Figure 9).^191^ In preparing their dimers, the Sun group elected to convert the N-terminal amine of demethylvancomycin into the corresponding azide to facilitate dimerization via triazole formation with a variety of bis-alkynes. In addition, a lipophilic group was appended to the vancosamine (Vv) site. The dimers this formed were found to have no enhancement of potency against MRSA and *S. pneumoniae* (MIC = 6.25–25 μg/mL), whereas against VRE the activity of dimer **34** did exceed that of demethylvancomycin by ≥2–4 fold.^191^

In another recent report describing glycopeptide dimers, Herczegh and co-workers synthesized and characterized the first teicoplanin pseudo-aglycon *N,N*-terminal homodimers, **35** and **36** (Figure 9).^192^ As noted above, unlike vancomycin, teicoplanin does not exhibit cooperative dimerization as part of its mechanism of action. The lack of dimerizing activity for teicoplanin is hypothesized to be due to the presence of the large acyl tail appended to the amino sugar at position 4 (Figure 1), which is speculated to anchor in the bacterial membrane and make binding to nascent lipid II more favorable.^29,30^ Herczegh and colleagues therefore hypothesized that, by removing this hydrophobic moiety and covalently linking the corresponding pseudo-aglycon, the resulting dimers could have improved activities.^192^ To this end, two strategies were employed: In the first, the teicoplanin pseudo-aglycon, lacking the carbohydrate at position 4 and bearing a C-terminal diethylaminopropylamide, was dimerized via a PEG linker featuring a lipophilic substituent to yield analogue **35**. In the second strategy, a histidine residue was first coupled to the N-terminus of the teicoplanin pseudo-aglycon lacking the carbohydrates at amino acid 4 and 7, followed by coordination with a simple Co^3+^ Schiff base complex to form the dimeric species **36**.^192^ Disappointingly, dimers **35** and **36** both showed diminished potency against MRSA (MIC = 4 μg/mL) when compared to teicoplanin (MIC = 0.5 μg/mL).^192^ Only against a VanA-type VRE strain did the activities of **35** and **36** improve, with MICs of 4–8 μg/mL relative to that of teicoplanin (MIC = 256 μg/mL).^192^ Although derivatives **34**–**36** show improved activities against VRE strains compared to their respective parent compounds, these N-terminal dimers are not as potent against MRSA when compared to the C-terminally linked homodimers of Sharpless^190^ and Haldar^188^ (**32** and **33**), highlighting the importance of the ligation site for antibacterial activity.

### Targeted Glycopeptide Delivery

**Figure 10:**
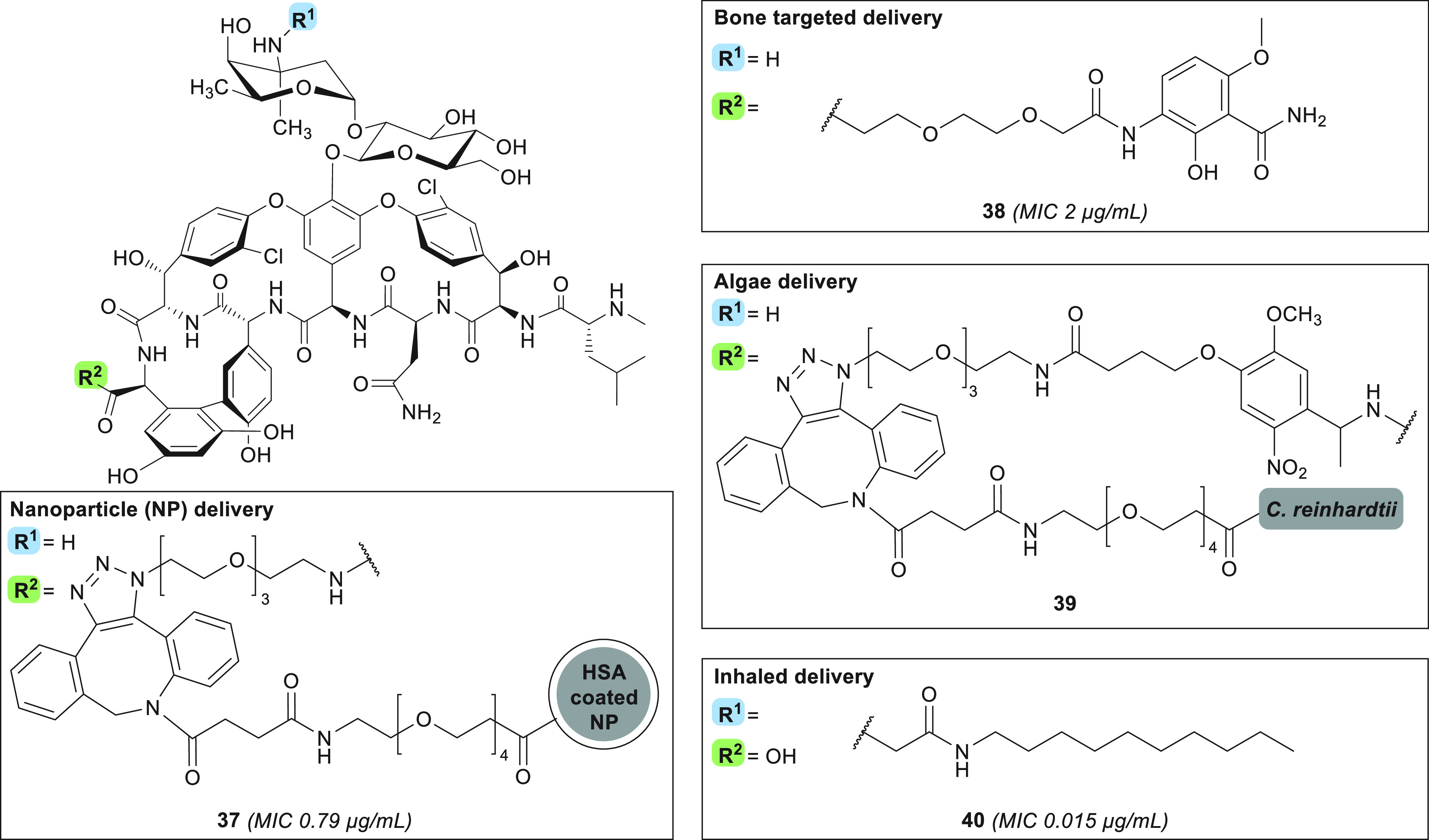
Glycopeptides designed for targeted drug delivery. MIC values are relative to experiments carried out on MRSA strains.

Glycopeptide antibiotics are generally administered systemically, potentially leading to unwanted side effects and to the development of resistant strains. To overcome these issues, efforts directed toward delivering vancomycin and its analogues in a targeted and controlled fashion have been reported in recent years. In this context, the use of technologies such as liposomes^193,194^ and dendrimers^195^ has been investigated. In addition to these non-covalent drug delivery systems, progress has also been made in covalently loading vancomycin on dendrimers or metal nanoparticles (NPs).^196-−199^ Cooper and colleagues conjugated an *N-*hydroxysuccinimide (NHS)-activated PEG-dibenzocyclooctyne (DBCO) to a human serum albumin monolayer bound to the surface of super-paramagnetic carboxylated 170 NPs.^200^ Subsequently, the NPs were loaded with vancomycin-PEG-N_3_ at different densities, using a copper-free azide–alkyne cycloaddition reaction, yielding derivative **37** (Figure 10). Low-density **37** was found to retain potent activity against MRSA (MIC = 0.79 μg/mL), and high-density **37** exhibited an 18-fold improved activity compared to vancomycin against VanA/B-type VRE (MIC = 28.9 μg/mL).^200^ The improved *in vitro* antibacterial potency of these nanoparticle-bound vancomycin derivatives is ascribed to two factors: (1) the enhanced binding affinity of **37** to the bacteria’s cell surface (for high density particles), highlighted by the fact that antagonization of bacterial inhibition requires a 64-fold molar excess of acetyl-Lys-d-Ala-d-Ala, and (2) the membrane permeabilization properties of **37**, which lead to membrane rupture for all density particles at 10-fold MIC.^200^

In addition to NP conjugation for improved drug delivery, vancomycin has also been modified with substituents designed to direct targeting to specific tissues and organs. The development of such approaches is of particular interest for those indications where vancomycin is advised as a first-line treatment, such as for targeting the bones in treating osteomyelitis, the skin for SSSIs, and the lungs in case of pulmonary infections. In one such strategy to specifically tackle osteomyelitis, for which *S. aureus* is a leading cause,^201^ researchers at the University of Louisville coupled a functional group with known hydroxyapatite affinity and enhanced bone accumulation abilities to the vancomycin C-terminus (compound **38**, Figure 10).^202^ Given vancomycin’s poor distribution to the skeletal tissue, the local concentration of the therapeutic agent at the target site is low, and prolonged administration is required, diminishing efficacy and increasing the potential for resistance development.^201,203^ By comparison, compound **38** was found to maintain *in vitro* antibacterial activity against MRSA (2 μg/mL)^202^ and in rats has a 1-log-reduced MRSA titer in an osteomyelitis model compared to the same dosing of vancomycin.^204^ Localization of **38** to the target site was confirmed in rats, with ∼5-fold higher concentrations in the bone compared to vancomycin after 12 h and 47-fold higher after 168 h. However, this particularly long exposure time can also lead to adverse events such as renal toxicity and leukocytosis.^203,204^

In 2020, Gademann and co-workers developed a light-irradiation-triggered release system by functionalizing the surface of *Chlamydomonas reinhardtii* with vancomycin, specifically aimed at SSSI treatment, as local and light-triggered release was hypothesized to minimize resistance selection.^205^ This living functionalized algae carrier was chosen as it is biodegradable^206^ and does not trigger immune response in mice,^207^ and chemical engineering of the surface had been demonstrated previously.^208^ The algae were functionalized using the well-established DBCO handle, allowing for copper-free azide–alkyne cycloaddition. Vancomycin was modified at the C-terminus via the installation of a PEG spacer containing the photocleavable *o*-nitrobenzyl moiety and a terminal azide handle. The azide-modified vancomycin species was subsequently conjugated to the DBCO-decorated algae, resulting in species **39** (Figure 10).^205^ While the covalent linkage of vancomycin to the algae surface was demonstrated to prevent the antibiotic from exerting its antimicrobial effect, upon light irradiation and subsequent linker cleavage, **39** was shown to inhibit growth of *B. subtilis* at both the lag phase (at 2.5 μM loading) and the exponential phase (at 5 μM loading) (MIC = 0.06 μg/mL), with release of free vancomycin-NH_2_ upon UV irradiation of **39** also confirmed.^205^ In order to establish the clinical potential of delivery system **39** for the intended SSSI treatment, it will need to be further assessed against relevant pathogens for this disease profile, such as *S. aureus* and β-hemolytic streptococci.^209^

In addition to the treatment of osteomyelitis and SSSIs, vancomycin is also used as a front-line therapy for persistent pulmonary MRSA infections. The drawbacks associated with vancomycin therapy for this indication, which requires high-dose systemic administration, include insufficient accumulation in the lungs and risk of renal toxicity. To address this, the group of Konicek set out to design derivatives of vancomycin suitable for inhalation.^210^ These analogues resemble telavancin but contain a carbonyl linker at the vancosamine position and no resorcinol modification. Representative amide **40** (Figure 10) was selected for extensive investigation due to (1) its potent *in vitro* activity against target bacteria MRSA (MIC = 0.015 μg/mL), *S. pneumoniae* (MIC = 0.008 μg/mL), *C. difficile* (MIC = 0.015–0.06 μg/mL), VanA-type VRE (MIC = 0.03–2 μg/mL), and VanB-type VRE (MIC = 0.03 μg/mL) (Table 1) and (2) its prolonged exposure time after inhalation in rats, with a half-life of 108 h, minimal conversion to the hydrolysis product, and minimal systemic toxicity.^210^ Amide **40** was also found to have enhanced anti-biofilm activity compared to vancomycin. Furthermore, nebulized **40** was assessed in an *in vivo* acute pulmonary MRSA infection model in neutropenic rats, where it demonstrated antibacterial activity that was superior to that of inhaled vancomycin.^210^ Overall, targeted glycopeptide strategies do show promise; however, care and attention are required to ensure that such constructs are tailored to have optimal PK profiles that allow them to reach their designated specific target sites while displaying minimal systemic toxicity.

### Glycopeptides Active against Gram-Negative Bacteria

**Figure 11:**
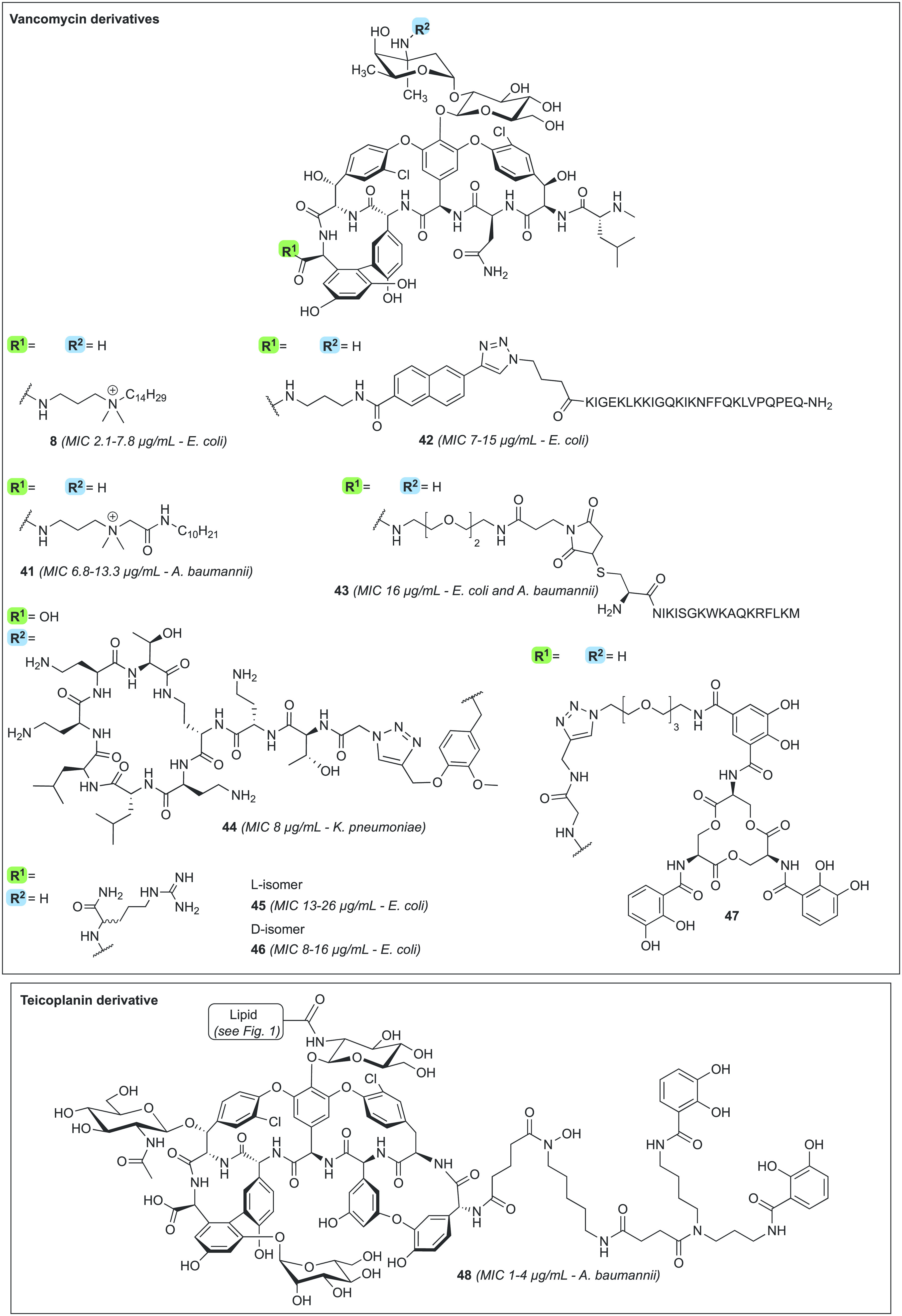
Glycopeptides with activity against Gram-negative bacteria.

Although most semisynthetic glycopeptide antibiotics target Gram-positive strains, the primary target of this class of antimicrobial agents—lipid II—is also present in Gram-negative bacteria. Vancomycin and other glycopeptides are inactive against Gram-negative bacteria due to their inability to cross the outer membrane (OM). However, the ability of vancomycin to bind to *E. coli*’s lipid II has been established previously.^220^ Potentiation of vancomycin by OM disruption by means of serum supplementation^221^ or the addition of synergists as adjuvants has also been demonstrated.^222,223^ While co-administration of lipopolysaccharide (LPS)-active OM disruptors potentiates vancomycin, these agents can also be covalently linked to the glycopeptide (Figure 11). In this regard, the previously discussed lipophilic cationic vancomycin analogue **8** was further investigated for activity against Gram-negative strains. The *in vitro* potency of **8** was assessed, where it showed moderate activity against *E. coli* (MIC = 2.1–7.8 μg/mL) and *Acinetobacter baumannii* (MIC = 5.2–9.0 μg/mL), as well as *K. pneumoniae* (MIC = 15.6 μg/mL) and MDR *Pseudomonas aeruginosa* (MIC = 10.6 μg/mL) (Figure 11, Table 2).^211^ The efficacy of this vancomycin derivative is reduced 2-fold in the presence of bovine serum albumin, likely due to its lipophilic nature and the consequent high protein binding.^211^ Notably, the anti-*A. baumannii* activity was also demonstrated in an *in vivo* murine thigh infection model, where compound **8** was found to reduce the bacterial titer by 3-log compared to vehicle. Building upon these findings, the Haldar group went on to design derivative **41**, containing an amide bond between the lipid and ammonium moiety envisioned to engage in additional hydrogen bonding. This semisynthetic vancomycin derivative was found to have activity against a panel of *A. baumannii* clinical isolates (MIC = 6.8–13.3 μg/mL) (Figure 11, Table 2).^212^ Furthermore, when administered at 50 μM, compound **41** reduces *A. baumannii* biofilm thickness in a concentration-dependent fashion, with 4–5-fold thinner biofilm formed compared to both vancomycin-treated and untreated biofilms. The results of subsequent *in vivo* experiments also indicate that the inclusion of the extra amide functionality improves the toxicity profile compared to **8** when administered IV. Furthermore, no propensity for resistance selection against *A. baumannii* was observed for either **8** or **41**.^211,212^ Mechanistically, both of these compounds are thought to inhibit cell-wall biosynthesis and exhibit outer and inner membrane permeabilization of both exponential and stationary phase cells, for which the permanent positive charge carried by the ammonium moiety appears essential.^211^ Like **8**, vancomycin analogue **41** retains *in vitro* activity against MRSA (0.7 μg/mL) while also showing activity against VISA (0.17 μg/mL) and VRE (MIC = 3.8–6.9 μg/mL).^212^

**Table 2:** *In Vitro* Antibacterial Activity against Gram-Negative Strains^a^

		MIC (μg/mL)				
category	compound	*E. coli*	*K. pneumoniae*	*A. baumannii*	*P. aeruginosa*	refs
Gram-negative active	**8**	2.1–7.8	15.6	5.2–9.0	10.6	(211)
	**41**	22–43	>173	6.8–13.3	22 to >173	(212)
	**42**	7–15	nd	nd	nd	(213)
	**43**	16	64	16	128	(214)
	**44**	16	8	32	16–64	(215)
	**45**	13–26	nd	51	103	(216)
	**46**	8–16	nd	8–32	nd	(217)
	**47**	nd	nd	nd	nd	(218)
	**48**	>128	nd	1–4	>128	(219)

a nd = not determined.

Following similar approaches, the van der Eycken and Huang groups independently reported the conjugation of lysine-rich antimicrobial peptides to the vancomycin C-terminus.^213,214^ The resulting derivatives **42** and **43** (Figure 11) were envisioned to cause OM disruption by interfering with divalent cation binding of LPS. While both compounds displayed reduced potency against the Gram-positive *S. aureus* (8–30 μg/mL),^213,214^ their ability to target Gram-negative strains is noteworthy. Analogue **42** was shown to be active against *E. coli*, *Yerisina enterocolitica*, *Pseudomonas putida*, and *Salmonella typhimurium* (MIC ≤4–30 μg/mL) (Table 2), for which anti-biofilm activity was also established (IC_50_ = 4–8 μg/mL).^213^ Compound **43** displays significant enhancement in antibacterial activity (MIC = 16 μg/mL) compared to vancomycin (MIC > 128 μg/mL) against *E. coli* and *A. baumannii* (Table 2).^214^ The enhanced activity of **43** toward Gram-negative species indeed appears to be the result of an OM-specific effect, given that the compound showed no reduction in cell viability in mammalian cell lines.^214^

In 2021, our team developed a panel of OM-disrupting vancomycin derivatives by linking the known OM disruptor and LPS-binder polymyxin E nonapeptide (PMEN) to the C-terminus or vancosamine portion of vancomycin using CuAAC conjugation.^215^ These derivatives, termed the vancomyxins, show improved *in vitro* potency compared to vancomycin alone or vancomycin supplemented with PMEN against Gram-negative bacterial strains. For example, derivative **44** (Figure 11) exhibited MIC values against *K. pneumoniae* and *E. coli* of 8 and 16 μg/mL, respectively (Table 2).^215^ The activity of the vancomyxins was also shown to be antagonized by LPS, suggesting that they do exert their activity via LPS binding, with OM disruption contributing to their mode of action due to the conjugation to PMEN.^215^ Besides showing activity against a panel of Gram-negative strains, and contrary to analogues **42** and **43**, vancomyxins such as **44** retain potent activity against a variety of Gram-positive bacteria, including MRSA (MIC = 0.25 μg/mL) and VRE, for which an up to 16 000-fold improvement compared to vancomycin was measured.^215^ Compound **44** displays no hemolysis and has a TD_50_ of 0.23 mM in proximal tubule epithelial cells, a concentration several orders of magnitude higher than the corresponding MIC values.^215^

While the analogues described above are the result of extensive structural modifications, small adjustments to vancomycin can also enhance activity against Gram-negative bacteria. During their studies on octaarginine conjugation via the C-terminus, culminating in vancomycin analogue **15**, Wender and Cegelski serendipitously discovered derivatives **45** and **46**, featuring the presence of a single l/d-arginine amide at the same position (Figure 11).^216,217^ Compounds **45** and **46** were found to display activity against Gram-negative bacteria (Table 2), including against MDR *E. coli*, with MIC values of 13–26^216^ and 8–16 μg/mL,^217^ respectively. Moreover, derivative **46** was also shown to have activity against some *A. baumannii* species (MIC = 8–32 μg/mL).^217^ These conjugates retain activity against Gram-positive isolates, prove non-hemolytic, and notably cause little permeabilization of the OM.^216^ The authors attribute the anti-Gram-negative activity of **45** and **46** to their ability to displace the LPS-stabilizing Mg^2+^ cations, a feature which is usually linked to self-promoted uptake.^216^ Furthermore, the *in vitro* activity of **46** was reflected *in vivo*, where it reduced the *E. coli* thigh burden in a murine model in a dose-dependent manner (4- to 7-log greater reduction compared to vancomycin or vehicle). Also of note is the finding that the relatively small structural difference between analogue **46** and the parent antibiotic results in an increased half-life in mice (1.29 h versus 0.89 h for vancomycin).^217^

Another strategy to transport glycopeptide antibiotics to their target site is facilitating active transport across the OM by covalent linkage to siderophores. Siderophores are iron-chelating agents produced by microorganisms to sequester iron from the microenvironment. After binding iron, siderophores are trafficked back into the bacterial cell through dedicated transporters, after which they release the iron, which is used in key cellular processes.^224^ These iron uptake pathways have also been hijacked by microorganisms in generating a class of naturally occurring Trojan horse antibacterial agents known as the sideromycins. Sideromycins are siderophore-conjugated antibiotics that are actively transported past the OM through siderophore uptake receptors and into the bacterial cell whereby they can elicit their antibacterial effect.^224^ This strategy has inspired several research groups to design semisynthetic glycopeptide-based sideromycins with anti-Gram-negative activity. The first vancomycin-containing sideromycin was reported by Miller and co-workers in 1996.^225^ More recently, the group of Nolan used CuAAC to connect enterobactin, a triscatecholate siderophore with unparalleled affinity for iron,^226−228^ to the C-terminus of vancomycin.^218^ The resulting conjugate **47** (Figure 11) was shown to inhibit the growth of siderophore-deficient *E. coli* and *P. aeruginosa*. Given that the cargo size of compound **47** was deemed too large for active uptake, its antibacterial effect was ascribed to extracellular iron chelation and nutrient deprivation.^218^ Miller and co-workers also employed a similar strategy in developing bis-catechol/mono-hydroxymate teicoplanin analogues such as compound **48**, wherein the siderophore was introduced at the N-terminus (Figure 11).^219^ Compound **48** exhibited *in vitro* antibacterial activity against *A. baumannii* (MIC = 1–4 μg/mL), with impressive activity against a carbapenemase-positive strain (MIC = 1 μg/mL) (Table 2).^219^ Also of note, while **48** was found to retain some potency against Gram-positive *S. aureus* (MIC = 4 μg/mL), its anti-Gram-negative activity appears specific for *A. baumannii*, as it had no impact on *E. coli* and *P. aeruginosa* proliferation.^219^ In summary, conjugating cationic groups or siderophores to glycopeptides is a viable strategy to make Gram-negative strains more susceptible to this class of antibiotics, although the resulting MIC values usually still fall in the “intermediate activity range”.

## Conclusion and Perspectives

In order to address resistance to glycopeptides like vancomycin, much effort has been applied in designing semisynthetic analogues of natural occurring glycopeptides. As opposed to total synthesis, semisynthetic approaches are more time- and cost-effective and have already resulted in the introduction of three novel glycopeptide antibiotics to the clinic. While these glycopeptides display enhanced potency, telavancin (**3**) has a black box warning due to its associated toxicity,^93,98^ and dalbavancin (**4**) and oritavancin (**5**) have unusual PK properties owing to their extremely long half-lives.^113,115,135,136^ While this can be considered a feature in that it allows for simplification in dosing regimen,^113,115,135,136^ it also carries the risk that any adverse reaction may persist for weeks post treatment. Moreover, *in vivo* exposure to sub-therapeutic levels of these antibiotics can also confer selection for resistant sub-populations.^110,111,135,138^ Thus, there remains a need for novel glycopeptide antibiotics with both improved potencies and enhanced PK and safety profiles.

This Review highlights recent developments in the field of semisynthetic glycopeptides. In addition to covering new glycopeptides with enhanced activity against Gram-positive bacteria, we also summarize recent efforts at extending the activity of these antibiotics toward Gram-negative organisms. Also of note are recent reports describing glycopeptide analogues as a starting point for the design of novel antiviral agents (against, for example, influenza or COVID-19) as well as in the development of innovative diagnostics probes.^16,229−232^ Most research on semisynthetic glycopeptide derivatives revolves around the modification of vancomycin at one or more of the following sites: the vancosamine (Vv), C-terminus (Vc), N-terminus (Vn), and resorcinol (Vr). To date, a limited number of studies have attempted to elucidate which modification site gives the most potent analogues, revealing a subtle interplay between the nature and the positioning of the substituent(s) and their impact on antibacterial activity.

The majority of the strategies employed toward the development of novel glycopeptide antibiotics relies on enhancing the bacterial cell surface binding, which often translates into the design of glycopeptide derivatives containing additional positively charged groups. Not only has this approach proven successful in tackling Gram-positive bacteria, but it can also confer activity against Gram-negative strains. While in Gram-positive strains the presence of positively charged moieties on the antibiotic molecule is presumed to favorably impact the interaction with the negatively charged membrane, the precise mechanism by which this phenomenon occurs is yet to be explored in depth. In Gram-negative strains, the antibiotic’s cationic portions likely displace the LPS-stabilizing divalent cations, thus disrupting the OM.^215,216^ While the exogenous supplementation of vancomycin with positively charged small-molecule or peptide-based synergists is an established strategy to enhance its anti-Gram-negative activity,^223^ many of the derivatives presented in this Review provide evidence for the advantage of covalently linking the glycopeptide to a cationic OM-disrupting moiety. Covalent conjugation may facilitate co-localization to the bacterial cell surface, thus bringing the glycopeptide structure in close proximity to its target. Also of note is the fact that minor structural modifications of the cationic portion—as small as a single guanidine moiety or arginine amide—have the power of conferring enhanced potencies against Gram-positive bacteria and, in some cases, Gram-negative strains.^163,216,217^ Furthermore, lipidated moieties, alone or in combination with cationic substituents, have been widely demonstrated to improve antibacterial activity against resistant strains. Glycopeptides with such hydrophobic substituents are assumed to have the ability to anchor in the membrane and have been shown to depolarize or permeabilize the bacterial membrane.^88,125,127−129,142,145−148,151^ Also of note are recent studies elaborating the mechanism of semisynthetic glycopeptides by the introduction of groups aimed at bacterial targets other than the traditional Lipid II d-Ala-d-Ala termini. Such strategies include conjugation to pyrophosphate-targeting groups or linking to antibiotics with alternative targets, both of which have shown promise.^173,175,178,185^ Moreover, the covalent dimerization of glycopeptide antibiotics,^187,188,190−192^ inspired by vancomycin’s natural cooperative dimerization, can result in enhanced surface binding due to co-localization to the target site.^28−30^ Finally, while the introduction of additional carbohydrate units has also been explored primarily to address PK and toxicity issues, such modifications have also been found to result in improved target binding to d-Ala-d-Lac, likely facilitated by the introduction of favorable hydrogen-bonding interactions.^141,143,156^

In an effort to confer selectivity to glycopeptide antibiotics and to minimize their toxicity, targeted approaches have been investigated wherein conjugation to large systems (nanoparticles or living organisms such as algae) or specific tissue-targeting moieties allows for preferential delivery to the target site.^200,202,205,210^ In addition, exploitation of specific Gram-negative bacterial uptake receptors has also been investigated through the conjugation of glycopeptides antibiotics to siderophores.^218,219,225^ As different bacteria employ a multitude of different siderophore transporters, this approach has the potential to generate species- or even strain-selective antibiotics.

Overall, while a large number of promising new semi-synthetic glycopeptides have been described in recent years, the characterization of most remains limited to preliminary studies of *in vitro* potency and cell-based toxicity. In order for these new glycopeptide antibiotics to progress toward clinical trials and eventually into the clinic, further investigations and additional translational studies showing an improved therapeutic window compared to the currently clinically used glycopeptides will be necessary. Despite these challenges, the broad collection of potent semisynthetic derivatives disclosed in the literature since 2014 provides a source of optimism for the discovery of tomorrow’s antibiotics. As this overview shows, while the low-hanging fruit in antibiotic discovery may have been plucked a long time ago, judicious semi-synthetic modifications of glycopeptides still hold great promise as a means of further optimizing and expanding the clinical relevance of this important class of antibacterial agents.
